# Somatic activating mutations in *MAP2K1* cause melorheostosis

**DOI:** 10.1038/s41467-018-03720-z

**Published:** 2018-04-11

**Authors:** Heeseog Kang, Smita Jha, Zuoming Deng, Nadja Fratzl-Zelman, Wayne A. Cabral, Aleksandra Ivovic, Françoise Meylan, Eric P. Hanson, Eileen Lange, James Katz, Paul Roschger, Klaus Klaushofer, Edward W. Cowen, Richard M. Siegel, Joan C. Marini, Timothy Bhattacharyya

**Affiliations:** 1 Section on Heritable Disorders of Bone and Extracellular Matrix, National Institute of Child Health and Human Development, National Institutes of Health, Bethesda, MD 20892 USA; 2 Clinical and Investigative Orthopedics Surgery Unit, National Institute of Arthritis and Musculoskeletal and Skin Diseases, National Institutes of Health, Bethesda, MD 20892 USA; 3 Program in Reproductive and Adult Endocrinology, Eunice Kennedy Shriver National Institute of Child Health and Human Development, National Institutes of Health, Bethesda, MD 20892 USA; 4 Biodata Mining and Discovery Section, Office of Science and Technology, National Institute of Arthritis and Musculoskeletal and Skin Diseases, National Institutes of Health, Bethesda, MD 20892 USA; 5 Ludwig Boltzmann Institute of Osteology at the Hanusch Hospital of WGKK and AUVA Trauma Center Meidling, 1st Medical Department Hanusch Hospital, UKH Meidling, Kundratstr. 37, Vienna, 1120 Austria; 6 Immunoregulation Section, Autoimmunity Branch, National Institute of Arthritis and Musculoskeletal and Skin Diseases, National Institutes of Health, Bethesda, MD 20892 USA; 7 Immunodeficiency and Inflammation Unit, Autoimmunity Branch, National Institute of Arthritis and Musculoskeletal and Skin Diseases, National Institutes of Health, Bethesda, 20892 USA; 8 Office of the Clinical Director, National Institute of Arthritis and Musculoskeletal and Skin Diseases, National Institutes of Health, Bethesda, MD 20892 USA; 9 Dermatology Branch, National Institute of Arthritis and Musculoskeletal and Skin Diseases, National Institutes of Health, Bethesda, MD 20892 USA; 10 Present Address: Molecular Genetics Section, Medical Genomics and Metabolic Genetics Branch, National Human Genome Research Institute, National Institutes of Health, Bethesda, MD 20892 USA

## Abstract

Melorheostosis is a sporadic disease of uncertain etiology characterized by asymmetric bone overgrowth and functional impairment. Using whole exome sequencing, we identify somatic mosaic *MAP2K1* mutations in affected, but not unaffected, bone of eight unrelated patients with melorheostosis. The activating mutations (Q56P, K57E and K57N) cluster tightly in the MEK1 negative regulatory domain. Affected bone displays a mosaic pattern of increased p-ERK1/2 in osteoblast immunohistochemistry. Osteoblasts cultured from affected bone comprise two populations with distinct p-ERK1/2 levels by flow cytometry, enhanced ERK1/2 activation, and increased cell proliferation. However, these *MAP2K1* mutations inhibit BMP2-mediated osteoblast mineralization and differentiation in vitro, underlying the markedly increased osteoid detected in affected bone histology. Mosaicism is also detected in the skin overlying bone lesions in four of five patients tested. Our data show that the *MAP2K1* oncogene is important in human bone formation and implicate MEK1 inhibition as a potential treatment avenue for melorheostosis.

## Abstract (web summary)

Melorheostosis is characterized by bone overgrowth and associated with pain and functional impairment. Here, the authors use whole exome sequencing to identify somatic mutations in *MAP2K1* in affected bone of melorheostosis patients which is associated with increased proliferation but delayed differentiation of cultured osteoblasts.

## Introduction

**Fig. 1:**
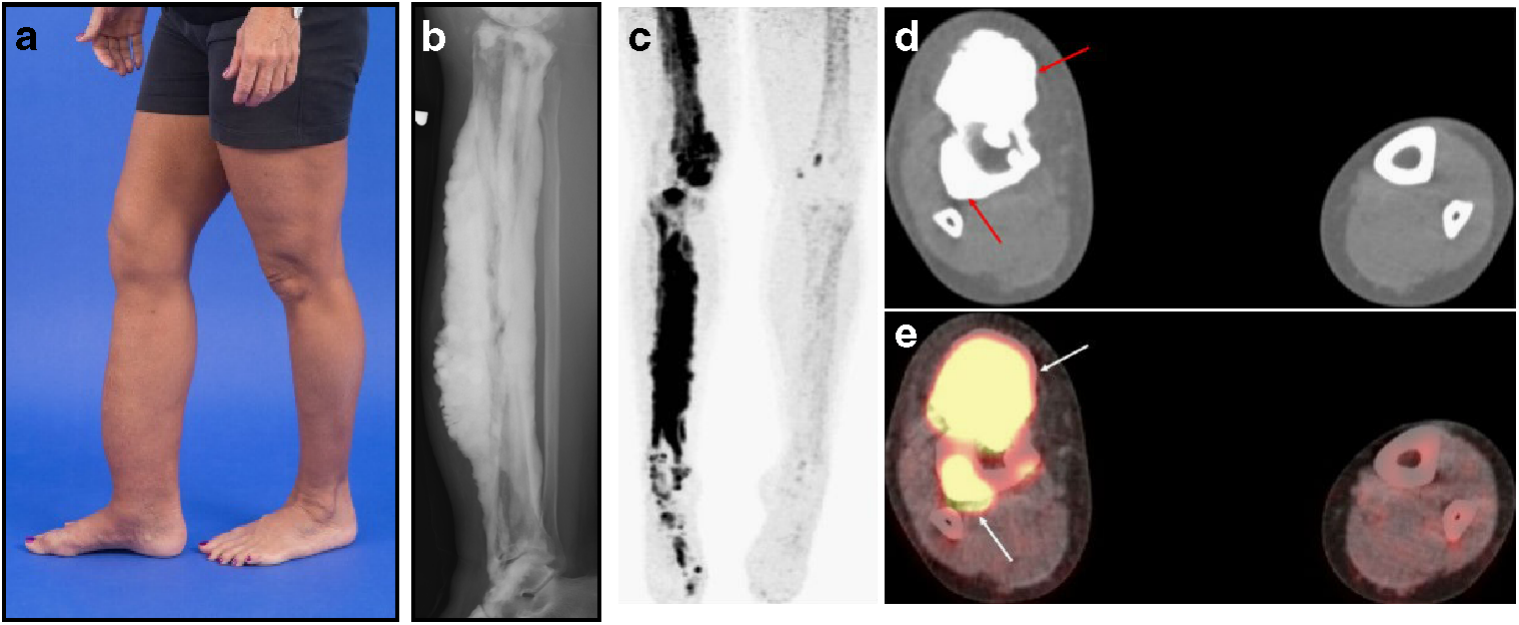
Clinical findings in melorheostosis. **a** Clinical appearance of Melo-10 with melorheostosis of right lower extremity. Note irregular thickening of the right leg. The affected bone was found to harbor a *MAP2K1* mutation. **b** Radiograph of the right tibia/fibula of Melo-10 with classic candle-wax appearance. **c** Maximal intensity projection (MIP) ^18^F-NaF PET image of the lower extremities showing intensely increased ^18^F-NaF activity in the bones of the right leg, primarily along the medial and distal femur, and along the tibia extending into the foot. **d**, **e** Axial CT and fused ^18^F-NaF PET/CT images at the level of mid-calf demonstrating dense cortical bone formation in the anterior and posterior aspect of the right tibia (red arrows in **d** with corresponding abnormally increased ^18^F-NaF uptake (white arrows, in **e**)

Melorheostosis (OMIM%155950) is a rare dysostosis characterized by excess bone formation in the classic “dripping candle wax” pattern on the surface of bone, revealed radiographically (Fig. 1a, b and Supplementary Fig. 1)^1^. The disease occurs in males and females equally, without familial clustering, and often affects multiple contiguous bones unilaterally in an asymmetric distribution^2^. Symptoms of melorheostosis may begin in childhood; the diagnosis is usually apparent by the age of 20 years^3^. The bone overgrowth lesions are associated with pain, functional impairment, joint contracture, and deformity. There is a wide spectrum of clinical appearance^2^ with skin lesions overlying the affected bone noted in up to 30% of patients. Bone lesions do not metastasize, but conversion to osteosarcoma has rarely been reported^4^. Increased uptake on bone scintigraphy suggests that melorheostotic bone is metabolically active (Fig. 1c–e). Patients are diagnosed by a combination of clinical findings, radiographs and bone scan. There is no definitive diagnostic test or specific treatment for melorheostosis^2^.

At present, two patterns of occurrence of melorheostosis have been differentiated by genetics. Patients with the autosomal dominant “spotted bone disease” osteopoikilosis and the related condition Buschke–Ollendorff syndrome (OMIM #166700) may display features of melorheostosis, and have been shown to have germline loss-of-function mutations in *LEMD3*, encoding the inner nuclear membrane protein MAN1^5^. *LEMD3* mutations have not been found in patients with the more common sporadic melorheostosis without osteopoikilosis^6^; the genetic cause of their condition is undefined^7^. Recently, a patient with melorheostosis and familial osteopoikilosis was found to have a germline *LEMD3* mutation and a somatic *KRAS* mutation in an overlying area of scleroderma-like skin^8^. Whether the *KRAS* mutation was causative for the bone findings in this one patient remains undetermined. The lack of vertical genetic transmission and asymmetric involvement suggests that sporadic melorheostosis may be caused by somatic mutations in bone-forming cells^9^, but bone tissue has not been previously investigated for somatic mutations in bone forming cells.

We biopsied affected and unaffected bone of 15 patients with melorheostosis and compared the tissue whole exome sequences for each patient. We identified mutations in the *MAP2K1* gene in affected, but not unaffected, bone of eight patients. *MAP2K1* encodes the protein MEK1, a kinase whose activity is modulated by its negative regulatory domain. The *MAP2K1* mutations identified in melorheostosis patients cause substitutions in two residues of the MEK1 negative regulatory domain. We present genetic, functional and histological data supporting the enhancement of MEK1 activity predicted by the location of the mutations and the causative role of the *MAP2K1* mutations in melorheostosis. This study provides evidence of a bone disease caused by mutations in *MAP2K1*.

## Results

### Identification of mosaicism for *MAP2K1*

**Fig. 2:**
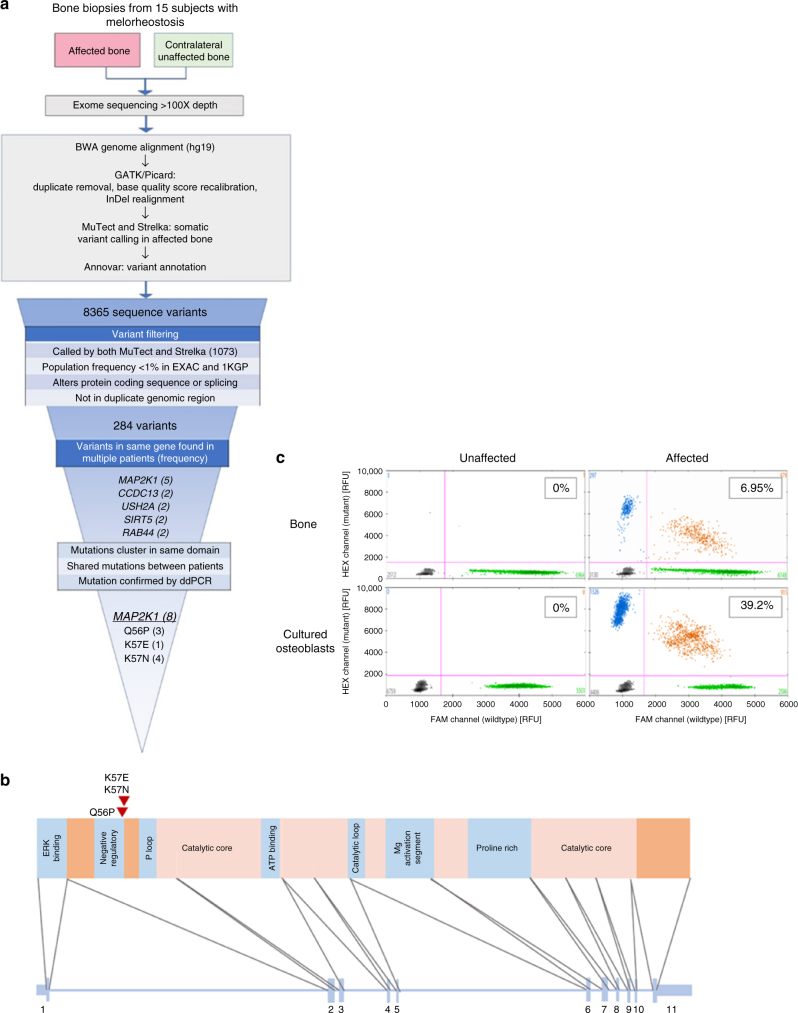
*MAP2K1* somatic mutations in melorheostotic bone. **a** Flowchart of genetic analysis. **b** Schematic of the *MAP2K1* exon structure encoding MEK1 protein domains. The three mutations from the eight patients are clustered in the negative regulatory domain. **c** Quantification of mutant allele abundance by ddPCR in unaffected and affected bone biopsies (top row), as well as in cultured osteoblasts from the respective biopsies (bottom row), from patient Melo-2 identified by WES to have a p.K57N mutation in affected bone. Each dot represents a droplet, with blue being mutant positive, green being wildtype positive, and orange being positive for both. Color-matched numbers correspond to count of droplets per quadrant. The boxed number is the fractional abundance of mutant allele in each sample

Fifteen patients with melorheostosis underwent biopsies of affected and unaffected bone (Supplementary Table 1 and 2). A consistent intraoperative finding was extremely dense, rigid affected bone that often dulled the osteotomes and drill bits. Genomic DNA was extracted from both tissues and subjected to high-depth whole exome sequencing. We identified 8365 sequence variants that were present in a subject’s affected bone but not unaffected bone. After restricting these variants to those that were found in more than 1% of sequences (but less than germline frequency), rare in the general population, and predicted to alter protein coding or mRNA splicing, we were left with 284 variants (Fig. 2a and Table 1). Only five genes contained putative somatic mutations in more than one affected bone sample (*MAP2K1*, *USH2A, CCDC13, SIRT5* and *RAB44*). Among these five genes, it was striking that sequences from five affected bone samples contained one of three tightly clustered missense mutations in the coding region of *MAP2K1*, c.167 A > C (p.Q56P), c.169 A > G (p.K57E), and c.171 G > T (p.K57N), encoding substitutions in two adjacent amino acids of the protein MEK1 (Fig. 2b and Supplementary Data 1). These coding variants are not present in the exomes of 123,136 healthy individuals searchable with the ExAC^10^. Both substituted residues are located in a MEK1 α-helix that negatively regulates kinase function (Fig. 2b and Supplementary Fig. 2)^11^. All three of these mutations have been previously shown to lead to gain-of-function through loss of the inhibitory role of the negative regulatory domain and have been identified in malignancies, including lung^12^, melanoma^13^ and hairy cell leukemia^14^ (Supplementary Table 3). All other 281 variants were unique to the affected bone of 1 of the 15 subjects (Supplementary Data 1). No germline or somatic *LEMD3*, *MAP2K2* or *MAP2K3* variants were identified in automated or manual re-examination of exome sequencing data from any patient. A *KRAS* p.Q61R mutation was found in both the affected and unaffected bone of one *MAP2K1* mutation-negative patient with skin lesions consistent with RASopathy, who likely has melorheostosis as part of a more complex early embryonic mosaic RASopathy, which will be reported separately. Thus 5 of 15 patients were identified to have *MAP2K1* mutations by whole exome sequencing.

**Table 1:** Table of individual patients showing the mutation location, cDNA and protein consequences, and variant allele frequencies (VAF)

Subject	AA Changes	VAF WES affected bone	VAF WES unaffected bone	VAF amplicon affected bone	VAF ddPCR affected bone	VAF ddPCR unaffected bone	VAF ddPCR skin	VAF ddPCR unaffected skin	VAF ddPCR blood
melo4	NM_002755:exon2:c.A167C:p.Q56P	10.1%^a^	0.00%	N.D.	9.4%	0.0%	12.5%	0.0%	0.0%
melo9	NM_002755:exon2:c.A167C:p.Q56P	17.19%^a^	0.00%	N.D.	20.0%	0.0%	N.D.	N.D.	0.0%
melo19	NM_002755:exon2:c.A167C:p.Q56P	30.86%^a^	0.99%	27.9%	27.8%	0.0%	N.D.	N.D.	N.D.
melo10	NM_002755:exon2:c.A169G:p.K57E	12.07%^a^	0.00%	16.7%	18.3%	0.0%	N.D.	N.D.	0.0%
melo2	NM_002755:exon2:c.G171T:p.K57N	6.09%	0.00%	7.2%	7.0%	0.0%	4.1%	0.0%	0.0%
melo6	NM_002755:exon2:c.G171T:p.K57N	3.12%	0.00%	4.8%	4.4%	0.0%	16.2%	0.0%	0.0%
melo16	NM_002755:exon2:c.G171T:p.K57N	1.04%	0.00%	2.7%	2.8%	0.0%	0.0%	0.0%	0.0%
melo18	NM_002755:exon2:c.G171T:p.K57N	25.21%^a^	0.0%	34.4%	33.6%	0.0%	6.5%	N.D.	0.0%

^a^ Cases where *MAP2K1* mutations were identified through automated filtering of WES for somatic mutations. Others were found by manual inspection. Mutations were not identified in normal control bone. Melo-19 had one mutant read in unaffected bone that was not seen in ddPCR, designating it as a sequencing error. The VAF results from ddPCR, amplicon and whole exome sequencing are highly correlated (*R* = 0.96 or greater for all comparisons, *p* < 0.003). *MAP2K1* mutations are present in overlying skin in four of five patients tested while contralateral skin is negative. N.D. not done

The allele frequency of these somatic heterozygous *MAP2K1* mutations identified by automated somatic mutation calling was relatively high (10–25%). We performed amplicon-based targeted sequencing to validate the exome sequencing findings and screen the remaining patients for *MAP2K1* mutations at higher sensitivity. This approach revealed three additional patients with *MAP2K1* p.K57N mutations at lower mutant allele frequencies (Table 1). Amplicon-seq polymerase errors were less than 1% at the base positions in question (Supplementary Tables 4, 5 and 6). Their mutations were missed by WES due to low allele frequencies in patients Melo-16 and Melo-6 (2.7% and 4.8%, respectively) or low coverage in patient Melo-2 (58×). All eight *MAP2K1* mutations were then confirmed by droplet digital PCR (ddPCR) of DNA from affected bone (Fig. 2c). The *MAP2K1* mutant allele frequency in affected bone by ddPCR ranged from 9 to 28% for p.Q56P, 3–34% for p.K57N, and 18% for p.K57E and correlates well with amplicon and WES data.

The ddPCR mutant allele frequency in osteoblasts cultured from melorheostotic bone of seven subjects, varied from 0.05 to 47% and was similar in different samples from the same cell culture, but often did not match the frequency found in bone tissue, consistent with tissue mosaicism. For example, there was a markedly higher frequency of the p.K57N mutation in cultured osteoblasts than bone tissue from patient Melo-2 (40–47% in cell passages 1–5 vs. 6.9% in bone) and a lower frequency of p.Q56P in Melo-4 (0.05–0.2% in cell passages 1–5 vs. 10% in bone tissue).

Mosaicism for their respective *MAP2K1* mutations was also detected in skin tissue overlying affected bone of patients Melo-2, Melo-4, Melo-6 and Melo-18, but not detected in skin from patient Melo-16, who has a lower disease burden. To look for germline mutations, we tested blood samples available from seven patients. *MAP2K1* mutations were not identified in blood samples including those with mutation-positive skin tissue, further confirming mosaicism (Table 1).

### Melorheostosis histology reveals intense remodeling

**Fig. 3:**
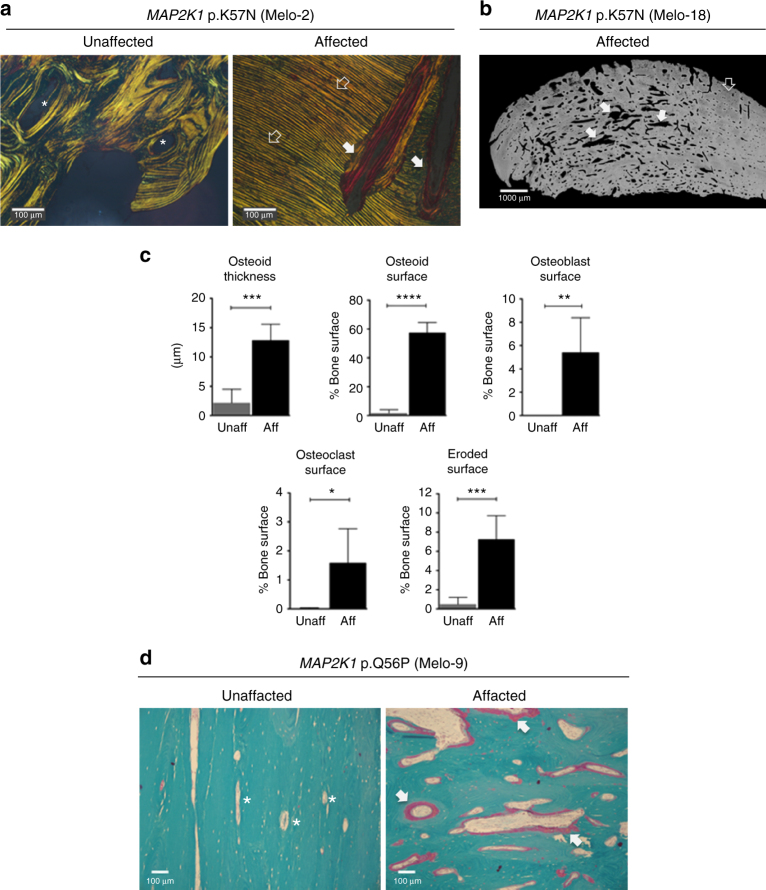
Intense bone remodeling and abnormal mineralization in melorheostosis. **a** Light microscopy images of cortical bone from patient Melo-2 showing the orientation of the collagenous matrix. Goldner’s trichrome staining is viewed under polarized light. Affected bone tissue shows in the periosteal regions appositional growth through deposition of layers of compact primary lamellar bone oriented parallel to the periosteal surface (open arrows). Subsequently these regions become highly remodeled. The solid arrows point towards large canals traversing the parallel layers of primary bone. Note the thick osteoid seams (red, unmineralized matrix) in affected bone tissue, whereas no osteoid is seen in unaffected bone (asterisks). **b** A backscattered electron image (BEI) of affected bone tissue from patient Melo-18. Gray represents the mineralized bone tissue, black is non-mineralized tissue. Note the extreme porosity of the bone tissue as a consequence of an intense bone remodeling activity (solid arrows). The open arrow is the same area labeled by the open arrow in **a**. **c** Bone histomorphometry. Comparison of indices of bone formation and bone resorption of affected vs. unaffected bone tissue from six patients with *MAP2K1* mutations. All indices of bone formation and bone resorption are significantly increased in affected bone tissue compared to unaffected bone of the same patient. *p < 0.05; **p < 0.01; ***p < 0.001; ****p < 0.0001 vs. values of unaffected bone tissue (paired *t*-test). Unaff: unaffected bone tissue, Aff: affected bone tissue. **d** Overviews of unaffected and affected bone tissue obtained from patient Melo-9. Affected bone can be easily recognized by its porosity and by the substantial portions of unmineralized matrix (solid arrows), while osteoid formation is not seen around osteonal canals in the unaffected bone sample (asterisks). Goldner’s trichrome staining followed by light microscopy in the bright-field mode. Green indicates mineralized bone matrix and red indicates unmineralized matrix

To gain insights into the effects of *MAP2K1* mutations on bone structure, remodeling and mineralization, we compared histomorphometry of tissue sections from affected and unaffected bone in six patients. Melorheostotic bone was characterized in the outer regions by distinctive parallel layers of primary lamellar bone, an organization that underlies the surgical hardness of the bone (Fig. 3a). This newly formed compact tissue was intensely remodeled into a highly porous osteonal-like bone at greater depth from the surface (Fig. 3b). Compared to unaffected bone, affected bone showed increased active bone-resorbing osteoclasts, elevated eroded surfaces and a higher number of bone-forming osteoblasts (Fig. 3c). There was an approximately six-fold increase in the thickness of osteoid (unmineralized bone matrix) and a greater than 50-fold increase in osteoid surface/bone surface in the affected bone samples compared to their respective unaffected counterparts (Fig. 3c, d).

### MEK1 signaling increased in melorheostosis

**Fig. 4:**
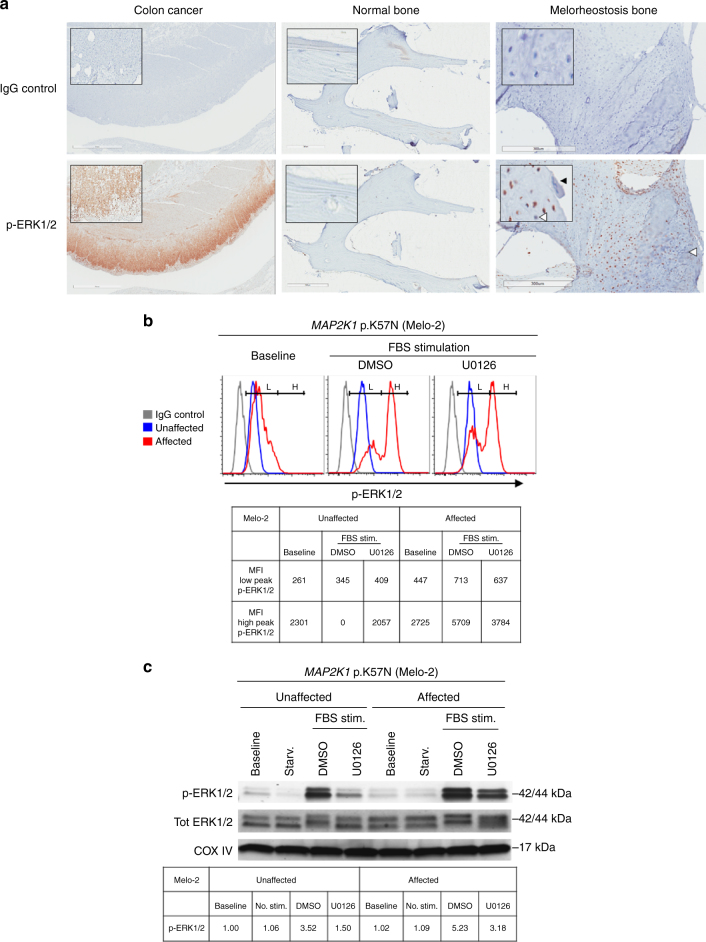
Activation of the MEK1-ERK1/2 pathway by *MAP2K1* mutations. **a** Immunohistochemical analysis of ERK1/2 activation in bone tissues from a melorheostosis patient. Left column shows sections of colon cancer stained with p-ERK1/2-specific antibodies. Marked brown staining is visible. Middle column shows a section of normal bone stained in a similar fashion. Right column shows representative section from melorheostotic bone (Melo-18, *MAP2K1* p.K57N, VAF 46%) stained with p-ERK1/2-specific antibodies. Osteocytes stain brown surrounded by woven bone. Cells positive for p-ERK1/2 are also seen in the periosteum. Inset shows high-power view of positively staining cells. A multinucleated osteoclast which does not stain for p-ERK1/2 is marked by the solid arrow. Cells negative for p-ERK1/2 are noted by the open arrows. See Supplementary Figure 3 for staining of SW48 colon cancer cells, which harbor the *MAP2K1* p.K56P mutation. **b** p-ERK1/2-specific flow cytometry analysis. Affected and unaffected osteoblasts from Melo-2 (*MAP2K1* p.K57N, VAF 45%) were stimulated with serum with or without MEK inhibitor U0126. Two peaks in the histogram indicates cell subpopulations of distinct level of p-ERK1/2 upon serum-stimulation in osteoblasts from affected bone (red), cells from unaffected bone (blue) only showed a single peak. U0126 reduced p-ERK1/2 in cells from affected bone. Cells stained with matching rabbit IgG isotype control are also shown (gray). The geometrical mean channel fluorescence is shown below for the high and low peaks of p-ERK1/2 marked on the histograms. **c** Western blot analysis of osteoblasts from affected and unaffected bone of patient Melo-2 (*MAP2K1* p.K57N, VAF 45%) shows increased ERK1/2 activation (p-ERK1/2) by MEK1 mutation in affected osteoblasts (lane 7 of p-ERK1/2 blot), as compared to unaffected osteoblasts (lane 3), by serum-stimulation. Inhibition of MEK1 with U0126 significantly diminished p-ERK1/2 in both affected and unaffected, but the level of p-ERK1/2 was still higher in affected osteoblasts compared to unaffected (lanes 4 and 8). Quantification data of band intensities are shown in a table. COX IV was used as control for equal amount protein loading

To determine how the *MAP2K1* mutations identified in our cohort affected the MEK1 target kinases ERK1 and ERK2, we examined MEK1 activity by immunohistochemical analysis of phosphorylated ERK1/2 (p-ERK1/2) in sections of affected bone tissue from two patients with *MAP2K1* mutations. Osteocytes and osteoblasts in affected bone displayed stronger p-ERK1/2 signals than control bone, but no p-ERK1/2 signal was detected in osteoclasts (Fig. 4a). Consistent with somatic mosaicism, the p-ERK1/2 positive cells occurred in patches in affected bone tissue.

Furthermore, osteoblasts cultured from affected bone and having a high frequency of mutant *MAP2K1* cells (approximately 80%) exhibited a bimodal ERK1/2 activation after serum-stimulation when examined by flow cytometry for intracellular p-ERK1/2. Bimodal ERK1/2 activation was not observed in cells from unaffected bone. Both peaks of ERK1/2 activation in osteoblasts from affected bone, likely representing wild-type and mutant cells, were reduced by the MEK-specific inhibitor U0126. Baseline p-ERK1/2 was also higher in a proportion of affected cells than in unaffected cells (Fig. 4b). These findings would be expected from a mixed cell population and confirm *MAP2K1* mosaicism at the cellular level.

We also investigated the MEK1-ERK1/2 pathway in cultured osteoblasts harboring *MAP2K1* mutations by western blot analysis in seven patients (Fig. 4c). Osteoblasts from the same patient and passage as those analyzed by flow cytometry showed increased p-ERK1/2 compared to cells from unaffected bone, indicating MEK1 gain-of-function (Supplementary Fig. 4). FBS-induced ERK1/2 activation could more readily be seen in unaffected osteoblasts by western blotting, likely due to the increased sensitivity in comparison to flow cytometry. Pre-treatment of cells with U0126 significantly diminished serum-stimulated p-ERK1/2, supporting enhanced MEK1 activity as mediating the increased ERK1/2 activation. The level of ERK1/2 activation by MEK1 generally correlated with mutant allele frequency. Activation was clearly seen in Melo-2 and Melo-18, with the highest percentage of mutant cells. In cells cultured from affected bone of Melo-10 with lower mutant allele frequency of 10%, the activation of ERK1/2 by MEK1 was equivocal (Supplementary Fig. 4).

### MEK1 activation increases cell proliferation

**Fig. 5:**
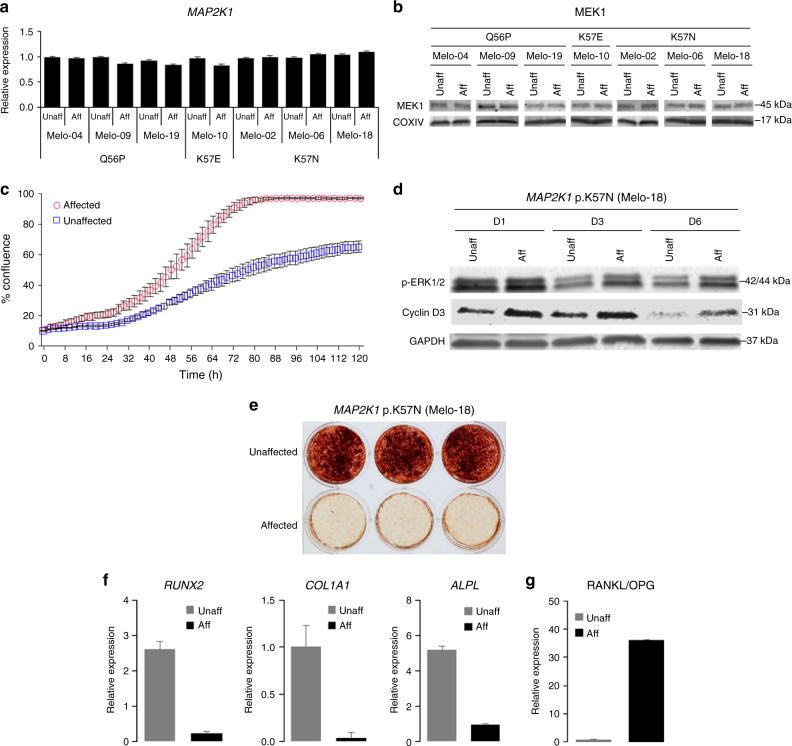
Increased cell proliferation and delayed osteoblast differentiation and mineralization by *MAP2K1* mutations. **a** *MAP2K1* variants found in seven melorheostosis patients do not affect the levels of *MAP2K1* transcripts. There was no statistically significant difference in *MAP2K1* transcript levels between affected and unaffected osteoblasts (*p *<* 0.05, paired *t*-test). Real-time qPCR was carried out in triplicate for each patient sample. **b** Western blot analysis displays comparable levels of MEK1 protein in affected and unaffected osteoblasts. **c** Cell proliferation assay using live-cell imaging. Affected and unaffected osteoblasts from melorheostosis patient, Melo-2 (*MAP2K1* p.K57N, VAF 45%) were plated at various densities (1000~5000 cells/well) (*n* = 30). Percent cell confluence is shown at 2-h intervals with symbols indicating mean of replicates (error bars: SEM). Doubling time calculated from the linear phase growth yielded a doubling time of 18 h for affected osteoblasts compared to 54 h for unaffected. Results are representative of two independent experiments. (*p* < 0.0001). **d** Western blot analysis shows that affected osteoblasts (lanes 2, 4, and 6) expressed higher level of cyclin D3 compared to unaffected (lanes 1, 3, and 5), correlating with the increased p-ERK1/2 level in affected cells shown in Fig. 4c. Note that levels of cyclin D3 and p-ERK1/2 decreased on day 3 (D3) and day 6 (D6) compared to day 1 (D1), because culture media was not refreshed after day 1, similar to conditions used during the live-cell imaging shown in Fig. 5c. **e** Alizarin Red S staining of mineralization in osteoblast cultures from patient Melo-18 (*MAP2K1* p.K57N, VAF 46%). After 7 weeks of BMP2-stimulated mineralization in vitro, significantly inhibited mineralization was observed in affected cells compared to unaffected. **f** Real-time qPCR analysis of expression of osteogenic marker genes, *RUNX2*, *COL1A1*, and *ALPL* in osteoblasts from melorheostosis patient Melo-2 (*MAP2K1* p.K57N, VAF 45%) after two weeks of osteogenic stimulation. Expression level of *RUNX2, COL1A1,* and *ALPL* was significantly lower in affected osteoblasts compared to unaffected. **g** The RANKL/OPG transcript ratio, an index of osteoclastogenic stimulus, was assessed by real-time qPCR with Melo-2 patient osteoblasts as in Fig. 5f. Significantly higher ratio of RANKL/OPG in affected osteoblasts indicates increased osteoclastogenesis compared to unaffected

*MAP2K1* mutations did not affect *MAP2K1* transcripts (Fig. 5a) or MEK1 protein levels (Fig. 5b), supporting a gain-of-function mechanism intrinsic to the protein itself. ERK1/2 activation promotes G1 to S-phase cell cycle progression and accelerates cell proliferation. The enhanced MEK1-ERK1/2 activation in affected osteoblasts increased cell proliferation in vitro (Fig. 5c). For example, the doubling time of osteoblasts from affected bone of patient Melo-2 (mutant allele frequency 45%) was 18 h, while osteoblasts from this patient’s unaffected bone had a doubling time of 54 h. Osteoblasts from affected bone did not lose contact inhibition. Furthermore, affected osteoblasts showed increased levels of cyclin D3 expression compared to unaffected cells, consistent with accelerated G1-S cell cycle transition (Fig. 5d).

### MEK1 activation decreases osteoblast differentiation in vitro

To further investigate the functional consequences of the *MAP2K1* mutations causing melorheostosis, osteoblast differentiation and mineralization were assessed in vitro. The presence of the *MAP2K1* mutation strikingly inhibited BMP2-stimulated mineralization in affected compared to unaffected osteoblasts (Fig. 5e). Expression of marker genes for osteoblast differentiation was also reduced in cells with the *MAP2K1* mutation during BMP2-stimulated differentiation (Fig. 5f). Reduced expression of *RUNX2* (runt-related transcription factor 2), *ALPL*(alkaline phosphatase) and *COL1A1* (the α1 chain of type I collagen*)* indicates the prevalence of immature bone forming cells. Osteoblasts early in differentiation secrete proteins to stimulate osteoclastogenesis^15^. In fact, we observed a markedly increased ratio of RANKL/OPG transcripts in osteoblasts from affected bone, which would generate a strongly positive context for osteoclastogenic stimulus (Fig. 5f)^16^.

Together, these data support key histological findings in affected bone. Inhibition of mineralization by overactive MEK1 accounts for the massive accumulation of unmineralized osteoid in affected bone tissue (Fig. 3d). Furthermore, the elevated RANKL/OPG ratio stimulates the increased osteoclast number and intense remodeling that occurs in melorheostotic bone (Fig. 3c).

## Discussion

Genetic osteosclerotic disorders are a rare but interesting subset of bone diseases which can provide important insights into bone biology. Identification of a germline mutation causing sclerosteosis led to the development of a potential treatment for osteoporosis^17–19^. Investigations into fibrodysplasia ossificans progressiva elucidated the role of inflammation in heterotopic ossification and exposed targets for treatment^20^. Here, our investigation has revealed a role for MEK1 in a disorder characterized by excessive bone formation.

We identified mutations in *MAP2K1* in the affected, but not unaffected, bone of 8 of 15 patients diagnosed clinically and radiologically with melorheostosis. The mutations cluster at two residues in the MEK1 negative regulatory domain, p.Q56 and p.K57, where identical substitutions have been identified in multiple malignancies, but not primary bone tumors. The occurrence of the mutations in a MEK1 “hot spot,” and the evidence of enhanced MEK1-ERK1/2 signaling in melorheostotic bone and osteoblasts, strongly support *MAP2K1* somatic mutations as causing about half of cases of sporadic melorheostosis. In our cohort, we found no occurrences of somatic or germline mutations in *LEMD3*, which have previously been reported in patients with familial osteopoikilosis and features of melorheostosis^6, 8^.

Mosaicism for cells with increased ERK1/2 activation was confirmed directly in melorheostotic bone by immunohistochemistry. Furthermore, two populations of cells with distinct levels of ERK1/2 activation were detected by flow cytometry. Increased ERK1/2 activation was also detected in cultured osteoblasts from affected, but not unaffected bone. In individual patients, the proportion of cultured mutant osteoblasts often differed from the proportion of mutant allele in genomic DNA extracted from bone chips from the same lesion, consistent with clustering of affected cells in mosaic conditions.

The three *MAP2K1* mutations we identified in melorheostosis have been shown to increase MEK1 activity in transfection studies^21^, and likely act by destabilizing the α-helix comprising the MEK1 negative regulatory region, which normally keeps the ATP-binding site in an inactive conformation^11^. MEK1 is a downstream activator in the RAS pathway. Mutations in RAS pathway components are associated with developmental defects falling under the umbrella designation RASopathies^22^ and involve variable cardiac, facial and neurodevelopmental defects. Interestingly, germline mutations identified in leukocytes from patients with the RASopathy cardio-facio-cutaneous syndrome^23, 24^ include two *MAP2K1* mutations (p.G128V/p.Y130C) in the catalytic core, and putatively activating mutations in the negative regulatory region of *MAP2K1* (p.F53S) or *MAP2K2* (p.F57V, analogous to *MAP2K1* p.F53). Bone overgrowth is not reported in these RAS-MAPK syndromes, implying that the somatic mutations we report in osteoblasts produce a different phenotype than the same mutation in the germline. Mosaicism for mutations at the same residues (p.Q56P and p.K57E) we identified in the MEK1 negative regulatory domain in melorheostosis patients has recently been reported in endothelial, but not skin or blood, cells of patients with extracranial arteriovenous malformations (AVM)^25^. Similar to melorheostotic bone lesions, AVM lesions caused by *MAP2K1* mutations expand but do not metastasize.

While the MAPK pathway is known to be involved in skeletogenesis and MEK1 is essential in embryogenesis, our study documents the importance of the MEK1-ERK1/2 pathway in human bone cell biology. While somatic mutations in *MAP2K1* have also been reported in Langerhans cell histiocytosis and Erdheim–Chester disease (ECD)^26^, the mutated cells in ECD are derived from monocytes rather than osteoblasts. Bone lesions in ECD are typically bilateral and symmetrical and occur in trabecular bone of the epiphysis^27^. In contrast, activation of ERK1/2 MAP kinases in melorheostosis occurs in osteoblast lineage and the lesions are primarily cortical.

Transgenic murine models, in which the entire MEK1 negative regulatory domain was deleted, share the elevated ERK1/2 signaling seen in melorheostosis^28^. However, the mice displayed accelerated bone remodeling rather than bone overgrowth. The mouse osteoblasts also showed increased differentiation and mineralization, but unchanged proliferation in vitro.

In melorheostosis, activation of the MEK1-ERK1/2 pathway in mature bone results in an increase in osteoblast surface and increased production of unmineralized bone matrix (osteoid). This is consistent with the faster proliferation of osteoblasts cultured from melorheostotic bone and with the less differentiated osteoblast phenotype revealed in vitro in BMP2-stimulated cells, in which transcripts of osteogenic genes *RUNX2*, *COL1A1*, and *ALPL* were decreased. Histologically, we observed a significant increase in osteoid formation and osteoclast numbers in affected bone from the *MAP2K1* variant cohort. Interestingly, these features were previously noted in a single case report^29^ with unknown genetics. The increased osteoid seen in vivo is consistent with the decreased mineralization deposited by affected osteoblasts in vitro with BMP2 stimulation. Increased osteoclast numbers and bone turnover in affected bone is consistent with the increased RANKL secretion by cultured cells, which would stimulate osteoclast development and intense bone remodeling. Furthermore, it is known that chronic activation of ERK1/2 strongly inhibits BMP2-mediated mineralization, as previously shown in a murine model for neurofibromatosis^30^, and provides an underlying mechanism for the increased unmineralized osteoid found in melorheostotic bone. The phenotypic distinction between murine models and melorheostosis patients may be ascribed to overexpression of the MEK1 transgene in all murine osteoblasts as opposed to the mosaic mutations in melorheostosis, or greater disruption of function by deletion of the full negative regulatory domain^28, 31^. Furthermore, studies in fruit flies and zebrafish indicate that activating *MAP2K1* mutations affect ERK1/2 activation differently depending on pathways intrinsic to the target tissue and developmental state^22^.

Identification of a somatic mosaic mutation in osteoblasts accounts for the unique clinical features of melorheostosis. The “dripping candle wax” pattern observed on radiographs is due to marked appositional bone growth followed by aggressive remodeling. The radiographic pattern of multiple contiguous bones affected while others are spared may be due to longitudinal expansion of mutant osteoblasts precursors along the axis of development (Supplementary Fig. 5)^32^. The marked variability in the pattern of bone lesions between patients is likely related to mutations arising during different points during development, with earlier mutations resulting in an increased burden of disease. Further studies will delineate the roles of specific *MAP2K1* mutations in different tissues at various developmental stages.

Melorheostosis can now be viewed as a genetically heterogeneous group of dysostoses. The melorheostosis seen in patients who have “spotted bone” osteopoikilosis is associated with a mutation in germline *LEMD3*^6^. Whyte et al. have shown that additional somatic mutations in *KRAS* (and possibly other genes) may play a role in the development of “dripping candle wax” bone lesions on radiographs in some patients in an osteopoikilosis kindred^8^. Patients who have sporadic melorheostosis are a distinct group and do not have germline^6, 33^, or as we now show, somatic mutations in *LEMD3*. It remains to be seen if the radiographic similarities in melorheostosis caused by *LEMD3* mutations and *MAP2K1* mutations are due to involvement of a common pathway with activation of SMADs, or are simply a similar clinical outcome.

Our finding that three of four patients demonstrated mutations in the overlying skin raises the possibility that testing of skin for *MAP2K1* mutations may be a diagnostic test for melorheostosis. However, if the skin sample is negative, bone biopsy may still be necessary to detect the mutation, especially in patients with a low mutant allele frequency. Although the causative gene is an oncogene, the clinical data from many years is reassuring to patients that malignant degeneration is rare^2, 4^.

Development of a diagnostic test is especially valuable because identification of somatic *MAP2K1* mutations in melorheostosis raises the possibility of inhibiting MEK1 to treat melorheostotic bone lesions. Several MEK1 inhibitors have already been developed for malignancies with *MAP2K1* mutations identical to those we identified in melorheostosis^34^. Because melorheostosis is one of few osteosclerotic bone-forming diseases, study of the MEK1-ERK1/2 pathway may lead to insights relevant to diseases where bone formation is impaired, such as delayed fracture healing and osteoporosis.

## Methods

### Patient cohort and bone sampling

We recruited 20 unrelated adults with a diagnosis of melorheostosis to a NIAMS Institutional Review Board-approved protocol (NCT02504879). Diagnoses were confirmed by radiographs and increased uptake on ^18^F-NaF bone scan (Fig. 1). Patients were excluded if pregnant or lactating, if under the age of 18, if actively infected or if unable to provide informed consent. Study subjects were not compensated for their participation. Fifteen patients consented to bone biopsies of affected and contralateral unaffected bone (see Supplementary Table 1 for clinical characteristics). Patients consented to publication of photographs.

Patients underwent open surgical biopsy of the affected bone under fluoroscopic guidance. The site of the biopsy of affected bone was chosen to minimize risk. Thus the most superficial area of melorheostotic bone was chosen. Under general anesthesia, the limb was exsanguinated and a tourniquet elevated (in all but one patient with proximal femur lesion). The bone was exposed and any thickened periosteum divided. Fluoroscopy was used to confirm the site of affected bone. Four drill holes were used to create a rectangle, and an osteotome used to connect the drill holes. The rectangular piece of cortical bone was then removed and sectioned in the operating room. The sections were placed immediately in cell culture media (for DNA extraction and osteoblast culture) or 70% ethanol for histology. Hemostasis was obtained with electrocautery and the wound closed in layers. A contralateral control sample of bone with no radiographic evidence of melorheostosis was also obtained.

For some patients, such as Melo-2 and Melo-18, abundant melorheostotic bone was available for evaluation. For other patients, only a small amount of affected bone could be safely sampled; priority was given to bone for DNA extraction followed by osteoblast culture over other studies. In all patients, the smallest informative sample of normal bone was taken to minimize morbidity. Thus, normal bone was often a limiting reagent. See Supplementary Table 2 for details on which patient samples underwent particular experiments.

### Additional tissue sampling

In our initial study design, we were concerned that surgical biopsy of melorheostosis (which had never been done before) could lead to worsening of the disease, infection, or other complication. Therefore, only the smallest amount of usable bone was collected. After a shared mutation was identified and the absence of surgical complications assured, four patients returning for a follow-up visit underwent 6 mm punch biopsy of skin overlying an area of affected bone, and a control biopsy of unaffected skin from the contralateral side. Seven of the eight patients with *MAP2K1* mutations agreed to have blood tested for mutations. DNA from peripheral blood leukocytes was tested by ddPCR for presence of the mutation.

### Genomic DNA extraction and mutation detection in bone tissue

Soft tissues and cartilage were manually dissected from affected and unaffected bone of each patient. Following repeated washes in phosphate-buffered saline (PBS) containing no Ca^2+^ or Mg^2+^ to remove marrow, mineralized tissues were minced using a rongeur and spring scissors in a sterile conical bottom glass tube (Roboz, Gaithersburg, MD).

Genomic DNA was isolated from minced bone chips following the manufacturer’s instructions (Gentra Puregene, Qiagen). In brief, minced bone samples were resuspended in cell lysis solution and digested with 0.2 mg/ml proteinase K at 55 °C for 12 h. Digested proteins were precipitated and removed by centrifugation at 14,000×*g* for 3 min. The cleared supernatant was transferred to a new tube and genomic DNA was precipitated with isopropanol and collected by centrifugation at 14,000×*g* for 5 min. The DNA pellet was washed once with 70% ethanol and air-dried at room temperature for 5 min. DNA was resuspended in DNA hydration buffer (10 mM Tris, 1 mM EDTA, pH 8.0) and its concentration was measured using a NanoDrop spectrophotometer (ThermoFischer Scientific).Genomic DNA samples underwent deep Whole Exome Sequencing (Otogenetics), with 107–270× coverage for each sample. Most exome targets (82%) had at least 50× coverage in each sample. Agilent SureSelect Human 51 Mbp All Exon kit was used for exome capture. Paired-end sequencing was performed on Illumina HiSeq sequencers. Sequencing reads were returned in FASTQ format and were aligned to Human Reference Genome Build 37 using Burrows–Wheeler alignment Tool. The standard PICARD-GATK pipeline was used to remove duplicate reads, refine alignment around indels, and recalibrate base quality scores. The resulting BAM files (one per sample) served as inputs to somatic mutation callers (Fig. 2a).

Two somatic mutation callers (muTect v1.1.7 and Strelka v1.0.14^35^) were used, with default parameters for WES data. Somatic variants called by either method were annotated with functional impact and population frequency using ANNOVAR. We filtered somatic variants for those that were not present in the unaffected bone, caused protein or ncRNA sequence changes or splicing changes, had less than 1% frequency in ExAC and 1000 Genomes Project databases, were not in a duplicated genomic region, and were called by both muTect and Strelka. Using this method, 284 putative somatic variants were identified in 15 affected bone samples (Supplementary Data 1 and Supplementary Fig. 2).

Exome sequencing was complemented by high-coverage amplicon sequencing with an average sequencing coverage of more than 10,000 × per sample. A targeted NGS amplicon library prep kit from Swift Biosciences (56 G Oncology) was used to screen patients for *MAP2K1* somatic mutations. The panel targets cancer-associated mutations in 56 genes including 5 mutation hotspot regions in *MAP2K1* (exons 2, 3, 6, 7, 11), as well as the RAS codon 12, 13 and 61 hotspot mutations. The amplicon libraries were sequenced on Illumina MiSeq and variants were called with LoFreq 2.1.2.

To validate and quantitate the allele frequency of somatic mutations in other tissues and cells cultured ex vivo, we utilized ddPCR (Fig. 2c). The approach determines the abundance of each allele using allele-specific hydrolysis probes (Supplementary Table 7) complementary to mutant allele (FAM-labeled) and wild-type allele (HEX-labeled) and is based on partitioning of each sample into >10,000 nanoliter-sized droplets, in which PCR is carried out. gBlock oligos of 200 bp bearing the mutation of interest were designed by Integrated DNA Technologies, Inc. (Coralville, IA) and used as mutation-positive controls. Reactions were performed on the QX200 Droplet Digital PCR System (Bio-Rad), using standard PCR cycling conditions based on manufacturer’s instructions (10-min enzyme activation at 95 °C, followed by 40 cycles of 30-s 94 °C denaturation and 1-min 55 °C annealing/extension, finishing with a 10-min enzyme deactivation at 98 °C). QuantaSoft software was used to quantitate the concentration of each allele as the number of copies per microliter, using a Poisson distribution model to account for the number of target copies per individual droplet. Fractional abundance of mutant allele was calculated as the concentration (copies per microliter) obtained in FAM channel divided by sum of concentrations of FAM and HEX channels.

### Bone histology and histomorphometry

Bone samples from a subset of 6 patients with *MAP2K1* mutations were embedded in polymethylmethacrylate and prepared for histomorphometric analyses using standard procedures^36^. For histological examinations, thin sections (4 µm) were cut from the tissue blocks with a hard tissue microtome (Leica SM2500, Leica Microsystems Nußloch GmbH), deplasticized with 2-methoxyethyl-acetate and stained with modified Goldner’s Trichrome^36^ (Fig. 3d). A light microscope equipped with a video camera (Zeiss Axiophot, Zeiss AxioCam, Oberkochen, Germany) was used to obtain digital images of the sections that were analyzed using NIH ImageJ software (version 1.63)^37^. Bone histomorphometric analyses were performed on 4 randomly chosen areas throughout each bone section (Fig. 3c). Because bone samples from the various skeletal sites contained predominantly compact/osteonal bone, only indices of bone formation and resorption were evaluated: osteoid thickness (O.Th), osteoid surface per bone surface (OS/BS), osteoblast surface per bone surface (Ob.S/BS), osteoclast surface per bone surface (Oc.S/BS) and eroded surface per bone surface (ES/BS). Bone lamellar organization was observed under polarized light.

Subsequently, the residual blocks were prepared for backscattered electron imaging by grinding and polishing to obtain plane parallel surfaces, then carbon coated (Fig. 3a, b). The entire cross-sectioned bone sample area was imaged with a spatial resolution of 1.8 µm per pixel using a field emission scanning electron microscope (FESEM) (Zeiss Supra 40, Oberkochen, Germany) equipped with a four-quadrant semiconductor backscatter electron detector. The FESEM was operated with electron energy of 20 keV. The gray levels reflecting the mineral/calcium content were calibrated by the material contrast of pure carbon and aluminum.

Statistical evaluation was performed with GraphPad Prism 6.0 (GraphPad Software, Inc., La Jolla, CA, USA). Comparison of histomorphometric indices were based on paired *t*-test comparing affected vs. unaffected tissue in each patient. Statistical significance was considered as *p* < 0.05.

### Cell culture

Primary osteoblasts were grown from freshly minced bone chips according to the method of Robey and Termine^38^. Bone chips (~ 1 mm) were incubated in alpha-minimal essential medium (α-MEM, Life Technologies) containing type II collagenase (200 U/ml, Worthington Biochemical Corp., Lakewood, NJ) for 2 h at 37 °C with gentle rocking. Bone chips were allowed to settle briefly, then collagenase solution was removed and washed twice with PBS. Bone chips were placed in T75 tissue culture flasks with α-MEM supplemented with 10% fetal bovine serum (Gemini Bio-Products, West Sacramento, CA) and antibiotics (penicillin (100 U/ml) and streptomycin (100 µg/ml), Thermo Fisher Scientific Inc.). Bone chips were incubated for 2–3 weeks at 37 °C/8% CO_2_, refreshing culture media every 3 days. Osteoblasts of passages 1–4 were used in this study.

Genomic DNA was extracted from cultured osteoblasts using the Gentra Puregene kit (Qiagen), as described above. The proportion of mutant *MAP2K1* allele in DNA from multiple cell passages and revivals was determined by ddPCR as above.

### Cell proliferation assay by live-cell imaging

Osteoblasts were plated at the indicated densities in 96-well tissue culture dish and imaged at 2 h intervals for 120 h using the IncuCyte ZOOM Kinetic Imaging System (Essen Bioscience) at 10× magnification (Fig. 5c). During the live-cell imaging, cells were incubated in 37 °C/8% CO_2_. Percent confluence was calculated from the percentage of the well area occupied by cells. Doubling time was calculated using GraphPad Prism 6.0 using non-linear regression. To analyze comparable populations of cells between groups, only wells with confluency at the first time-point of image acquisition within one half standard deviation of the mean were included in the analysis. Statistical comparison was done by two-way ANOVA.

### Gene expression in osteoblasts by real-time quantitative PCR

Total RNA was extracted from osteoblasts using the RNeasy mini kit (Qiagen) following the manufacturer’s instruction. RNA concentration was measured by the NanoDrop spectrophotometer. Synthesis of cDNA was performed using 1 µg of total RNA and the High‐Capacity cDNA Reverse Transcription Kit (Thermo Fisher Scientific), following the manufacturer’s instructions. Comparative real-time PCR was performed in triplicate with an Applied Biosystems Prism 7500 Fast Sequence Detection System using TaqMan universal PCR master mix, according to the manufacture’s protocol (Applied Biosystems Inc., Foster City, CA) (Fig. 5a, f, g). The TaqMan probes and primers were purchased from Applied Biosystems: Human *RUNX2* (Hs01047973_m1), *COL1A1* (Hs00164004_m1), *ALPL* (Hs01029144_m1), RANKL (*TNFSF11*, Hs00243522_m1), OPG (*TNFRSF11B*, Hs00900358_m1). Human *GAPDH* (Hs02786624_g1) and *TBP* (Hs00427620_m1) were used as endogenous controls for normalization. Levels of *MAP2K1*, *RUNX2*, *ALPL* and *COL1A1*, RANKL (*TNFSF11*), and OPG (*TNFRSF11B*) transcripts were determined using the 7500 Fast System SDS software version 1.3.1 (Applied Biosystems). Relative expression was calculated using the comparative ∆∆Ct method.

### Flow cytometric analysis for intracellular p-ERK1/2

Osteoblasts cultured from affected and unaffected bone of melorheostosis patients were serum-starved for 1 h and stimulated with 20% FBS with or without MEK inhibitor U0126 (10 μM) for 30 min. Osteoblasts were then harvested for intracellular staining with antibodies for p-ERK1/2. After fixation with 4% paraformaldehyde and permeabilization with ice cold methanol, cells were washed in PBS containing 1% BSA. Cells were stained for 60 min in the dark at room temperature with the following antibodies (Cell Signaling Technology, Cambridge, MA): Phospho-p44/42 MAPK (ERK1/2) (Thr202/Tyr204), (197G2), (clone E10) rabbit monoclonal antibody (Alexa Fluor 647^®^Conjugate) (#13148) (1:100 dilution) or rabbit IgG isotype control (Alexa Fluor^®^ 647 Conjugate) #3452). Cells were acquired on a FACSVerse™ flow cytometer (Becton Dickinson) and analyses for p-ERK1/2 levels were performed by using FlowJo^®^ software (Tree Star) on live gated cells (Fig. 4b). The gating strategy is shown in Supplementary Figure 7.

### Western blot analysis of MEK1 and ERK1/2 activation

Osteoblast lysates were analyzed by western blotting using standard methods and indicated antibodies (Fig. 4c, b, d). Briefly, osteoblasts from affected and unaffected bone of melorheostosis patients were grown in α-MEM supplemented with 10% FBS (Gemini Bio-Products, West Sacramento, CA) and antibiotics (penicillin (100 U/ml) and streptomycin (100 µg/ml), Thermo Fisher Scientific Inc.). Cells were seeded at 75,000 cells/well in 12-well tissue culture vessel containing 1 ml/well of culture media. On the next day, cells were pre-treated with U0126 (1, 4-diamino-2, 3-dicyano-1,4-bis[2-aminophenylthio] butadiene, a MEK1/2 inhibitor, for 1 h. Inhibitor concentration in culture was 10 µM (#9903, Cell Signaling Technology) or DMSO (vehicle control, Sigma) in serum-free α-MEM. Cells were then stimulated with FBS (20% in media) for 30 min before being lysed in cell lysis buffer (50 mM Tris (pH 8.0), 150 mM NaCl, 1% IGEPAL, 0.5% sodium deoxycholate, 0.1% sodium disodium sulfate, 1× protease inhibitor cocktail, and 1× phosphatase inhibitor cocktail). Cell lysates were cleared by centrifugation at 20,000×*g* for 20 min at 4 °C. Soluble cell lysates were mixed with SDS-PAGE sample buffer (50 mM Tris, pH 6.8, 2% SDS, 10% glycerol, 0.1% bromophenol blue, and 100 mM dithiothreitol) and denatured at 80 °C for 10 min. Proteins were resolved in 4–15% mini-PROTEAN precast gels (Bio-Rad, Hercules, CA) at 100 V for 90 min. The resolved proteins were transferred to nitrocellulose membranes (Bio-Rad) at 100 V for 90 min at 4 °C. The membranes were immersed in 5% bovine serum albumin in Tris-buffered saline containing 0.5% Tween-20 (TBS-T) for 1 h at room temperature to block non-specific binding of primary antibodies. Subsequently, the membranes were probed with the following antibodies (Cell Signaling Technology, Cambridge, MA): phospho-p44/42 MAPK (Erk1/2) (Thr202/Tyr204) (clone #D13.14.4E) rabbit monoclonal antibody (#4370, 1:2000), p44/42 MAPK (Erk1/2) (clone # L34F12) mouse monoclonal antibody (#4696, 1:2000), MEK1/2 rabbit antibody (#9122, 1:1000), cyclin D3 (clone #DCS22) mouse monoclonal antibody (#2936, 1:2000), GAPDH (clone #D16H11) rabbit monoclonal antibody (#5174, 1:1000), and COX IV (clone # 3E11) rabbit monoclonal antibody (#4850, 1:1000). After incubating membranes with primary antibodies for 16 h at 4 °C, membranes were washed in TBS-T three times for 5 min each at room temperature. Protein bands were visualized by incubating membranes for 1 h at room temperature with the following secondary antibodies (LI-COR Biosciences, Lincoln, NE): IRDye^®^ 680RD anti-rabbit IgG (#926-68071, 0.1 ug/ml) or IRDye^®^ 800CW anti-mouse IgG (#926-32210, 0.1 ug/ml). The membranes were washed in TBS-T three times for 5 min each at room temperature before scanning with Odyssey^®^ Infrared Imaging Systems (LI-COR Biosciences, Lincoln, NE). Uncropped western blots are available in Supplementary Figure 6.

### Immunohistochemistry for p-ERK1/2

Fresh bone tissue was fixed in 10% neutral buffered formalin, decalcified by formic acid, and embedded in paraffin. Thin sections (5 µm) were mounted onto tape adhesive slides. Following deparaffinization and rehydration with ethanol, endogenous peroxidase was blocked using 3% hydrogen peroxide/distilled water. Antigen retrieval was performed using a citrate buffer (BioGenex) for 10 s at 100 °C, then slides were cooled and rinsed in distilled water and Tris-buffered saline/Tween-20 (TBS-T). After blocking in 5% normal goat serum (Vector Labs), sections were incubated with anti-phospho-p44/42 MAPK ERK1/2 rabbit monoclonal antibody (Thr202/Tyr204; diluted 1:200, Cell Signaling Technology, USA #4376) overnight at 4 °C or monoclonal rabbit IgG isotype control. Subsequently, biotinylated goat anti-rabbit IgG (1:100, Vector Labs) was applied for 30 min, before slides were incubated with ABC Elite Standard (Vector Labs) for 30 min. p-ERK1/2 was visualized by a 5-min incubation in DAB Reagent (Sigma) and subsequent hematoxylin counterstain. Slides were dehydrated, cleared in xylene and mounted with coverslips with Permount. Paraffin embedded colon cancer cells (SW48, carrying a p.Q56P mutation in *MAP2K1*) was used as positive control^13^. Normal bone from patients undergoing surgical resection for unrelated indications served as negative control (Fig. 4a).

### Osteoblast mineralization and differentiation in vitro

Osteoblasts from affected and unaffected bone of patient Melo-18 were plated at passage 2 with 50,000 cells/well in 12-well plates and cultured to confluency in α-MEM supplemented with 10% FBS and antibiotics at 37 °C/8% CO_2_. Following confluence, osteoblasts were grown in osteoblast differentiation medium (50 µg/mL L-ascorbic acid, 10 nM dexamethasone, and 2.5 mM β-glycerophosphate), with or without 100 ng/mL recombinant BMP2 (#355-BM, R&D Systems, Minneapolis, MN), refreshing osteogenic media every 3 days for 7 weeks.

To visualize mineralization in osteoblast culture, cells were washed once with PBS and fixed with 4% paraformaldehyde for 30 min at room temperature. Fixed cells were washed three times with PBS and stained with 2% Alizarin Red S solution (pH 4.2) for 30 min at room temperature. Excess Alizarin Red S stain was removed with three distilled water washes. Cells were air-dried in the dark before imaging (Fig. 5e). During osteoblast mineralization in osteogenic medium, total RNA was collected at 2 weeks’ post-induction and used to examine gene expression by real-time quantitative PCR analysis.

### Data availabilty

The data that support the findings of this study are available from the corresponding author upon reasonable request.

## Electronic supplementary material

Description of Additional Supplementary Files(PDF 29 kb)

Supplementary Data 1(XLSX 55 kb)

Supplementary Information(DOCX 15884 kb)
