# Supplementary material for: Somatic activating mutations in MAP2K1 cause melorheostosis
Source: Nat Commun. 2018 Apr 11;9:1390. doi: 10.1038/s41467-018-03720-z (PMC5895796; doi:10.1038/s41467-018-03720-z)
Supplement: Supplementary file 1 — Description of Additional Supplementary Files(PDF 29 kb) [file 41467_2018_3720_MOESM1_ESM.pdf]

Description of Additional Supplementary Files

File Name: Supplementary Dataset 1

Description: Variants found on whole exome sequence of melorheostotic bone
