# Supplementary material for: Somatic activating mutations in MAP2K1 cause melorheostosis
Source: Nat Commun. 2018 Apr 11;9:1390. doi: 10.1038/s41467-018-03720-z (PMC5895796; doi:10.1038/s41467-018-03720-z)
Supplement: Supplementary file 3 — Supplementary Information(DOCX 15884 kb) [file 41467_2018_3720_MOESM3_ESM.docx]

**Supplementary Appendix**

**Somatic Activating Mutations in *MAP2K1* Cause Melorheostosis**

Kang *et al.*

# Supplementary Table 1. Clinical Features of Melorheostosis Patients

| **Patient** | **Age** | **Gender** | **Affected Side** | **Affected Extremity** | **Site of Affected Bone Biopsy** | **Site of Unaffected Bone Biopsy** | **Mutation Status** |
| --- | --- | --- | --- | --- | --- | --- | --- |
| Melo 1 | 25 | Female | Left | Lower | left tibia | right tibia | Negative |
| Melo 2 | 42 | Male | Right | Lower | right tibia | left tibia | Positive |
| Melo 4 | 42 | Female | Right | Lower | right ankle | left tibia | Positive |
| Melo 6 | 50 | Female | Right | Lower | right tibia | left tibia | Positive |
| Melo 8 | 53 | Female | Left | Lower | left first metatarsal | right tibia | Negative |
| Melo 9 | 59 | Female | Right | Upper | right humerus | left ulna | Positive |
| Melo 10 | 49 | Female | Right | Lower | right tibia | left iliac crest | Positive |
| Melo 11 | 66 | Male | Left | Lower | left proximal tibia | right iliac crest | Negative |
| Melo 12 | 29 | Female | Left | Lower | left proximal tibia | right iliac crest | Negative |
| Melo 13 | 56 | Female | Right | Lower | right femur | left tibia | Negative |
| Melo 16 | 28 | Female | Left | Lower | left middle cuneiform | right tibia | Positive |
| Melo 17 | 40 | Male | Right | Upper | right radius | left ulna | Negative |
| Melo 18 | 45 | Female | Left | Upper | left clavicle | right iliac crest | Positive |
| Melo 19 | 42 | Male | Right | Upper | right radius | right iliac crest | Positive |
| Melo 24 | 60 | Male | Left | Upper | left tibia | right iliac crest | Negative |

# Supplementary Table 2. Experimental Evaluation of Patient Samples

| **Subject** | **Gene variation** | **WES** | **ddPCR** | **Western Blot** | **FACS** | **Proliferation** | **Immunohistochemistry** | **Bone Histology** | **Histomorphometry** |
| --- | --- | --- | --- | --- | --- | --- | --- | --- | --- |
| **Melo-4** | *MAP2K1* P.Q56P | Yes | Yes | Yes | No | No | Yes | Yes | Yes* |
| **Melo-9** | *MAP2K1* P.Q56P | Yes | Yes | Yes | Yes | Yes | No | Yes | Yes |
| **Melo-19** | *MAP2K1* P.Q56P | Yes | Yes | Yes | Yes | Yes | No | Yes | Yes |
| **Melo-10** | *MAP2K1* P.K57E | Yes | Yes | Yes | Yes | Yes | No | Yes | Yes |
| **Melo-2** | *MAP2K1* P.K57N | Yes | Yes | Yes | Yes | Yes | No | Yes | Yes |
| **Melo-6** | *MAP2K1* P.K57N | Yes | Yes | Yes | No | No | No | Yes | No |
| **Melo-16** | *MAP2K1* P.K57N | Yes | Yes | No | No | No | No | Yes | No |
| **Melo-18** | *MAP2K1* P.K57N | Yes | Yes | Yes | Yes | Yes | Yes | Yes | Yes |

* Two samples of affected bone were available for Melo 4. Both were used.

# Supplementary Table 3. Conditions Where *MAP2K1* Mutations Previously Identified

| **Mutation** | **Conditions Associated** |
| --- | --- |
| K57N | Primary non-small cell lung cancer^1^, melanoma^2^, IGHV4-34-expressing hairy cell leukemia^3^, Langerhans cell histiocytosis^4^ |
| Q56P | IGHV4-34 expressing hairy cell leukemia^3^, lung adenocarcinoma^5^, Langerhans cell histiocytosis^6^, extracranial arteriovenous malformations^7^ |
| K57E | IGHV4-34-expressing hairy cell leukemia^3^, resistance to Vemurafenib in metastatic melanoma^8^, Langerhans cell histiocytosis^4^, extracranial arteriovenous malformations^7^ |

# Supplementary Table 4. Read Counts from the MAP2K1 Amplicon Sequencing for Position 66727451

| **Sample** | **Ref** | **Coverage** | **A** | **T** | **C** | **G** | **a** | **t** | **c** | **g** | **Sub** | **Ins** | **Del** | **VAF** |
| --- | --- | --- | --- | --- | --- | --- | --- | --- | --- | --- | --- | --- | --- | --- |
| melo10-aff | A | 11775 | 5866 | 6 | 20 | 1 | 5877 | 0 | 2 | 3 | 32 | 0 | 0 | 0.27% |
| melo11-aff | A | 3729 | 1851 | 4 | 11 | 0 | 1861 | 0 | 1 | 1 | 17 | 0 | 0 | 0.46% |
| melo12-aff | A | 7026 | 3508 | 2 | 3 | 0 | 3513 | 0 | 0 | 0 | 5 | 0 | 0 | 0.07% |
| melo13-aff | A | 7855 | 3918 | 1 | 4 | 3 | 3929 | 0 | 0 | 0 | 8 | 0 | 0 | 0.10% |
| melo16-aff | A | 6104 | 3049 | 0 | 3 | 1 | 3046 | 3 | 2 | 0 | 9 | 0 | 0 | 0.15% |
| melo17-aff | A | 5133 | 2561 | 1 | 3 | 2 | 2565 | 0 | 0 | 1 | 7 | 0 | 0 | 0.14% |
| melo18-aff | A | 3750 | 1871 | 0 | 5 | 0 | 1874 | 0 | 0 | 0 | 5 | 0 | 0 | 0.13% |
| melo19-aff | A | 14181 | 5110 | 2 | 1977 | 2 | 5111 | 0 | 1979 | 0 | 3960 | 0 | 0 | 27.92% |
| melo1-aff | A | 23177 | 11533 | 10 | 46 | 3 | 11578 | 1 | 1 | 5 | 66 | 0 | 0 | 0.28% |
| melo24-aff | A | 6504 | 3250 | 0 | 1 | 2 | 3249 | 0 | 0 | 2 | 5 | 0 | 0 | 0.08% |
| melo2-aff | A | 8476 | 4223 | 2 | 14 | 3 | 4229 | 0 | 0 | 5 | 24 | 0 | 0 | 0.28% |
| melo6-aff | A | 4750 | 2373 | 1 | 0 | 1 | 2372 | 1 | 0 | 1 | 4 | 0 | 0 | 0.08% |
| melo8-aff | A | 5805 | 2894 | 3 | 1 | 4 | 2900 | 1 | 0 | 1 | 10 | 0 | 0 | 0.17% |
| melo10-ctl | A | 12621 | 6294 | 4 | 12 | 5 | 6299 | 3 | 2 | 2 | 28 | 0 | 0 | 0.22% |
| melo18-ctl | A | 6219 | 3103 | 1 | 4 | 2 | 3108 | 0 | 0 | 1 | 8 | 0 | 0 | 0.13% |
| melo24-ctl | A | 7528 | 3758 | 2 | 1 | 3 | 3762 | 0 | 1 | 1 | 8 | 0 | 0 | 0.11% |
| melo2-ctl | A | 12028 | 5992 | 6 | 19 | 0 | 6006 | 3 | 0 | 2 | 30 | 0 | 0 | 0.25% |
|  |  |  |  |  |  |  |  |  |  |  |  |  |  |  |
| Upper case letters: read counts from plus strand | | | | | | | |  |  |  |  |  |  |  |
| Lower case letters: read counts from minus strand | | | | | | | |  |  |  |  |  |  |  |

# Supplementary Table 5. Read Counts from the MAP2K1 Amplicon Sequencing 66727453

| **Sample** | **Ref** | **Coverage** | **A** | **T** | **C** | **G** | **a** | **t** | **c** | **g** | **Sub** | **Ins** | **Del** | **VAF** |
| --- | --- | --- | --- | --- | --- | --- | --- | --- | --- | --- | --- | --- | --- | --- |
| melo10-aff | A | 11776 | 4893 | 6 | 10 | 984 | 4905 | 1 | 0 | 977 | 1978 | 0 | 0 | 16.80% |
| melo11-aff | A | 3729 | 1847 | 5 | 11 | 3 | 1859 | 1 | 0 | 3 | 23 | 0 | 0 | 0.62% |
| melo12-aff | A | 7026 | 3496 | 7 | 6 | 4 | 3509 | 2 | 0 | 2 | 21 | 0 | 0 | 0.30% |
| melo13-aff | A | 7855 | 3909 | 5 | 7 | 5 | 3926 | 2 | 0 | 1 | 20 | 0 | 0 | 0.25% |
| melo16-aff | A | 6104 | 3049 | 0 | 1 | 3 | 3051 | 0 | 0 | 0 | 4 | 0 | 0 | 0.07% |
| melo17-aff | A | 5133 | 2563 | 1 | 3 | 0 | 2565 | 0 | 0 | 1 | 5 | 0 | 0 | 0.10% |
| melo18-aff | A | 3750 | 1867 | 3 | 5 | 1 | 1873 | 1 | 0 | 0 | 10 | 0 | 0 | 0.27% |
| melo19-aff | A | 14181 | 7085 | 1 | 4 | 1 | 7090 | 0 | 0 | 0 | 6 | 0 | 0 | 0.04% |
| melo1-aff | A | 23175 | 11529 | 19 | 29 | 13 | 11567 | 4 | 1 | 13 | 79 | 0 | 0 | 0.34% |
| melo24-aff | A | 6504 | 3242 | 3 | 5 | 3 | 3250 | 1 | 0 | 0 | 12 | 0 | 0 | 0.18% |
| melo2-aff | A | 8476 | 4223 | 2 | 11 | 6 | 4229 | 1 | 1 | 3 | 24 | 0 | 0 | 0.28% |
| melo6-aff | A | 4750 | 2374 | 0 | 2 | 0 | 2373 | 1 | 0 | 0 | 3 | 0 | 0 | 0.06% |
| melo8-aff | A | 5805 | 2893 | 2 | 5 | 3 | 2900 | 0 | 0 | 2 | 12 | 0 | 0 | 0.21% |
| melo10-ctl | A | 12622 | 6290 | 5 | 17 | 3 | 6301 | 1 | 0 | 5 | 31 | 0 | 0 | 0.25% |
| melo18-ctl | A | 6219 | 3107 | 1 | 1 | 1 | 3107 | 1 | 0 | 1 | 5 | 0 | 0 | 0.08% |
| melo24-ctl | A | 7528 | 3754 | 3 | 6 | 1 | 3760 | 1 | 3 | 0 | 14 | 0 | 0 | 0.19% |
| melo2-ctl | A | 12027 | 5984 | 6 | 20 | 6 | 6002 | 1 | 1 | 7 | 41 | 0 | 0 | 0.34% |
|  |  |  |  |  |  |  |  |  |  |  |  |  |  |  |
|  |  |  |  |  |  |  |  |  |  |  |  |  |  |  |
| Upper case letters: read counts from plus strand | | | | | | | |  |  |  |  |  |  |  |
| Lower case letters: read counts from minus strand | | | | | | | |  |  |  |  |  |  |  |

# Supplementary Table 6. Read Counts from the MAP2K1 Amplicon Sequencing 66727455

| **Sample** | **Ref** | **Coverage** | **A** | **T** | **C** | **G** | **a** | **t** | **c** | **g** | **Sub** | **Ins** | **Del** | **VAF** |
| --- | --- | --- | --- | --- | --- | --- | --- | --- | --- | --- | --- | --- | --- | --- |
| melo10-aff | G | 11776 | 3 | 6 | 0 | 5884 | 7 | 3 | 0 | 5873 | 19 | 0 | 0 | 0.16% |
| melo11-aff | G | 3729 | 0 | 2 | 1 | 1863 | 1 | 0 | 0 | 1862 | 4 | 0 | 0 | 0.11% |
| melo12-aff | G | 7026 | 2 | 1 | 2 | 3508 | 1 | 2 | 2 | 3508 | 10 | 0 | 0 | 0.14% |
| melo13-aff | G | 7855 | 1 | 0 | 0 | 3925 | 1 | 2 | 0 | 3926 | 4 | 0 | 1 | 0.05% |
| melo16-aff | G | 6104 | 1 | 82 | 0 | 2970 | 0 | 81 | 0 | 2970 | 164 | 0 | 0 | 2.69% |
| melo17-aff | G | 5133 | 1 | 1 | 0 | 2565 | 0 | 0 | 0 | 2566 | 2 | 0 | 0 | 0.04% |
| melo18-aff | G | 3749 | 0 | 647 | 0 | 1229 | 0 | 642 | 0 | 1231 | 1289 | 0 | 0 | 34.38% |
| melo19-aff | G | 14181 | 0 | 3 | 0 | 7088 | 1 | 1 | 0 | 7088 | 5 | 0 | 0 | 0.04% |
| melo1-aff | G | 23175 | 2 | 6 | 2 | 11579 | 11 | 7 | 1 | 11567 | 29 | 0 | 0 | 0.13% |
| melo24-aff | G | 6504 | 0 | 2 | 1 | 3250 | 0 | 1 | 1 | 3249 | 5 | 0 | 2 | 0.08% |
| melo2-aff | G | 8477 | 0 | 306 | 2 | 3934 | 4 | 306 | 0 | 3925 | 618 | 0 | 0 | 7.29% |
| melo6-aff | G | 4750 | 2 | 114 | 0 | 2260 | 2 | 114 | 0 | 2258 | 232 | 0 | 0 | 4.88% |
| melo8-aff | G | 5804 | 1 | 0 | 0 | 2901 | 1 | 2 | 0 | 2899 | 4 | 0 | 0 | 0.07% |
| melo10-ctl | G | 12623 | 2 | 6 | 2 | 6304 | 8 | 1 | 1 | 6299 | 20 | 0 | 0 | 0.16% |
| melo18-ctl | G | 6219 | 0 | 1 | 0 | 3109 | 0 | 2 | 0 | 3107 | 3 | 0 | 0 | 0.05% |
| melo24-ctl | G | 7528 | 0 | 0 | 0 | 3764 | 2 | 3 | 0 | 3759 | 5 | 0 | 0 | 0.07% |
| melo2-ctl | G | 12028 | 0 | 0 | 2 | 6014 | 5 | 5 | 0 | 6002 | 12 | 0 | 0 | 0.10% |
|  |  |  |  |  |  |  |  |  |  |  |  |  |  |  |
|  |  |  |  |  |  |  |  |  |  |  |  |  |  |  |
| Upper case letters: read counts from plus strand | | | | | | | | |  |  |  |  |  |  |
| Lower case letters: read counts from minus strand | | | | | | | | |  |  |  |  |  |  |

# Supplementary Table 7. Probes Used for ddPCR

| **Mutation** | **Forward primer** | **Reverse primer** | **Mutant probe (FAM)** | **Wildtype probe (HEX)** |
| --- | --- | --- | --- | --- |
| **MAP2K1 Q56P** | CGAAAGCGCCTTGAG | AAGTCGTCATCCTTCAGTT | TCTGCTTCGGGGTAAGA | TCTGCTTCTGGGTAAGAAA |
| **MAP2K1 K57N** | CGAAAGCGCCTTGAG | AAGTCGTCATCCTTCAGT | CCCAGAATCAGAAGGTGG | CCCAGAAGCAGAAGGTG |
| **MAP2K1 K57E** | CGAAAGCGCCTTGAG | AAGTCGTCATCCTTCAGTT | TCTTACCCAGGAGCAGA | TCTTACCCAGAAGCAGAAG |

All ddPCR mutation probe assays were custom-designed. Sequences for forward primer, reverse primer, mutant-binding probe, and wildtype-binding probe are

#
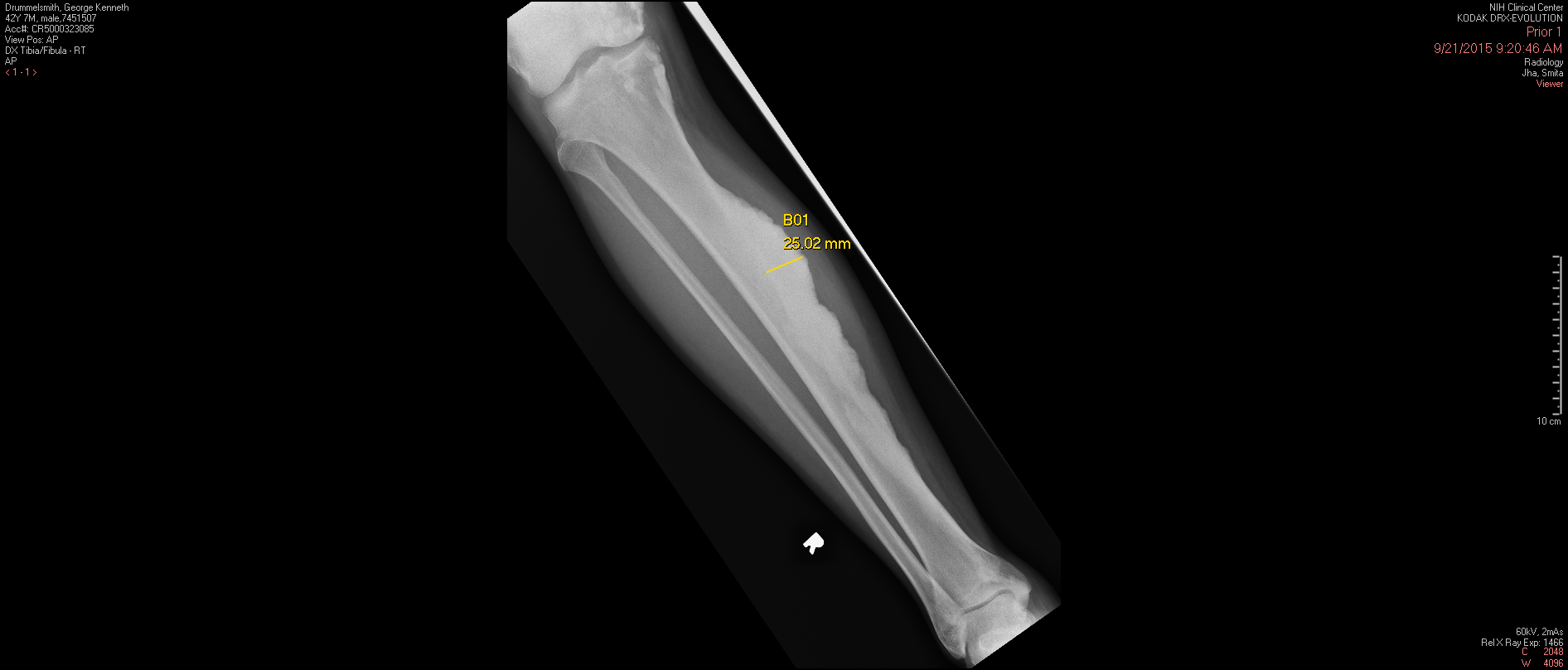
Supplementary Figure 1. Radiographs of Patients with *MAP2K1* Mutations


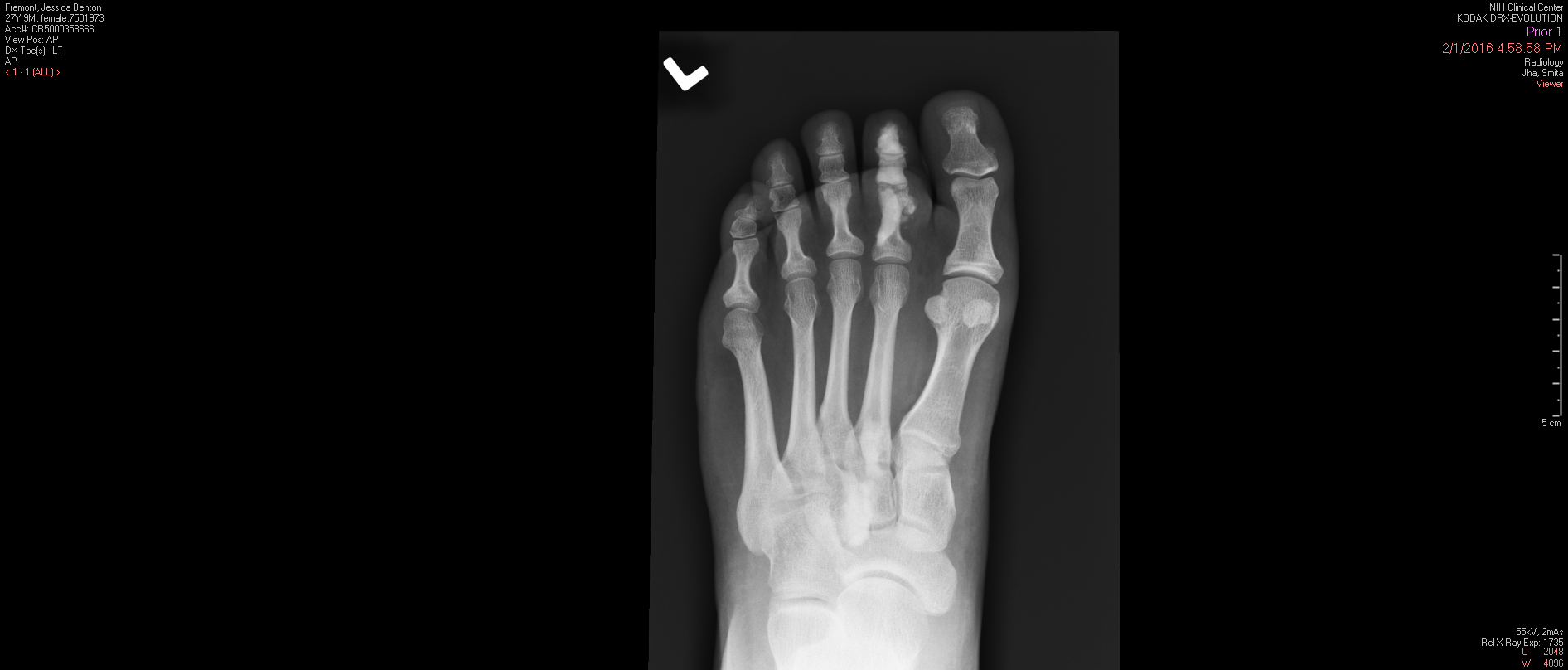

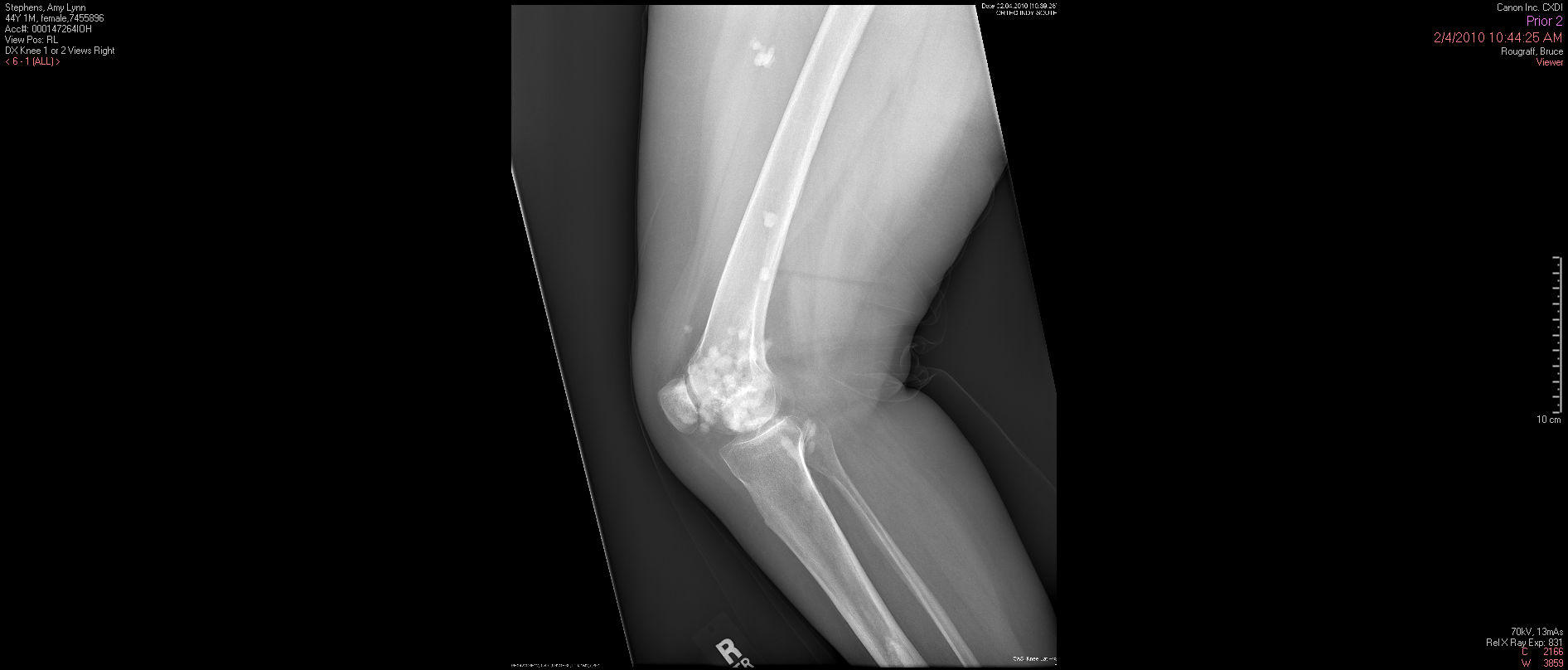

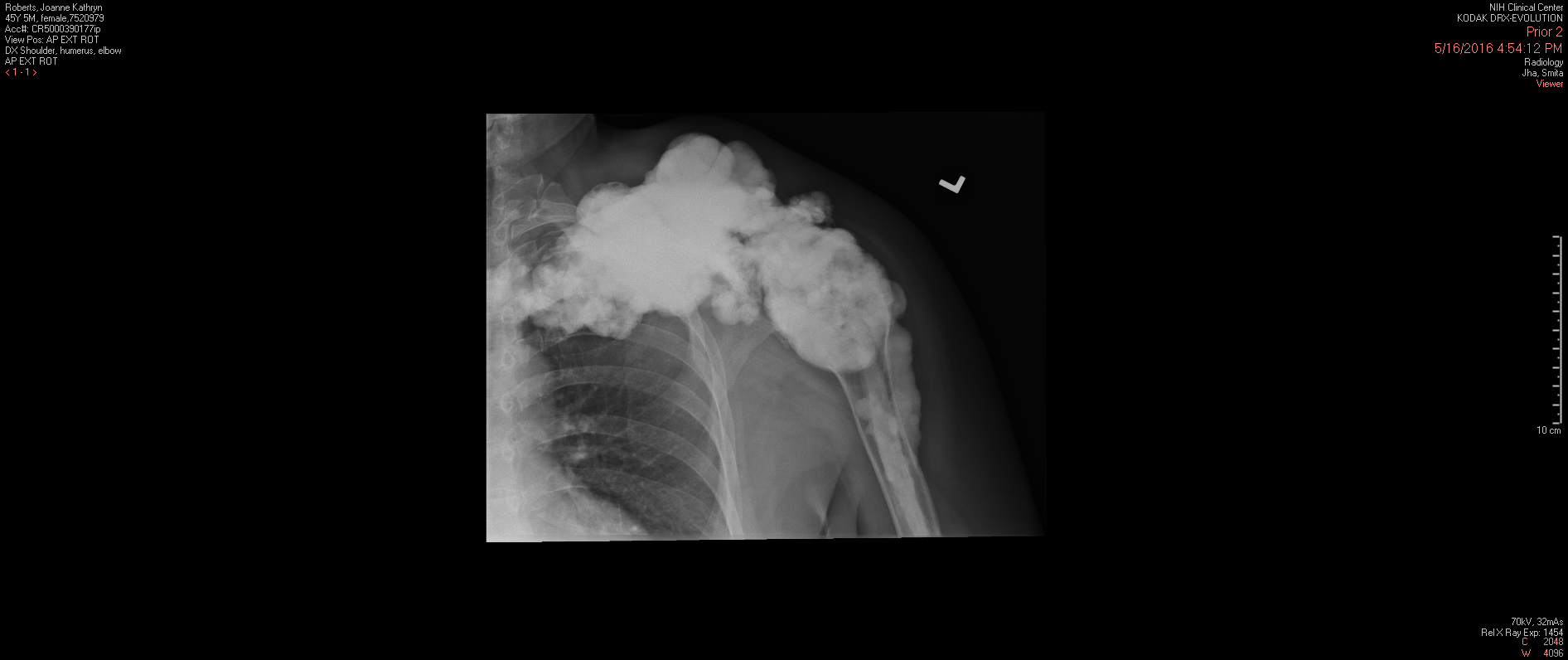


b

C


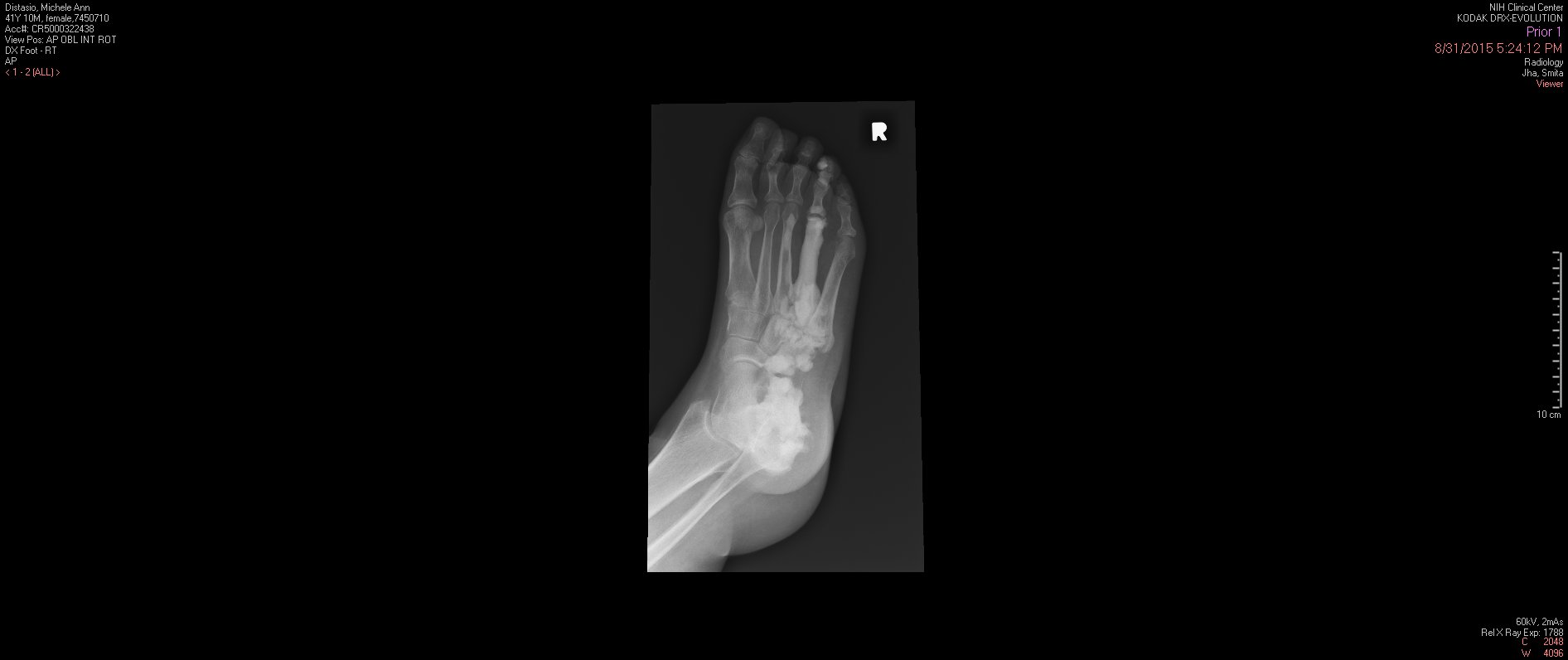


A


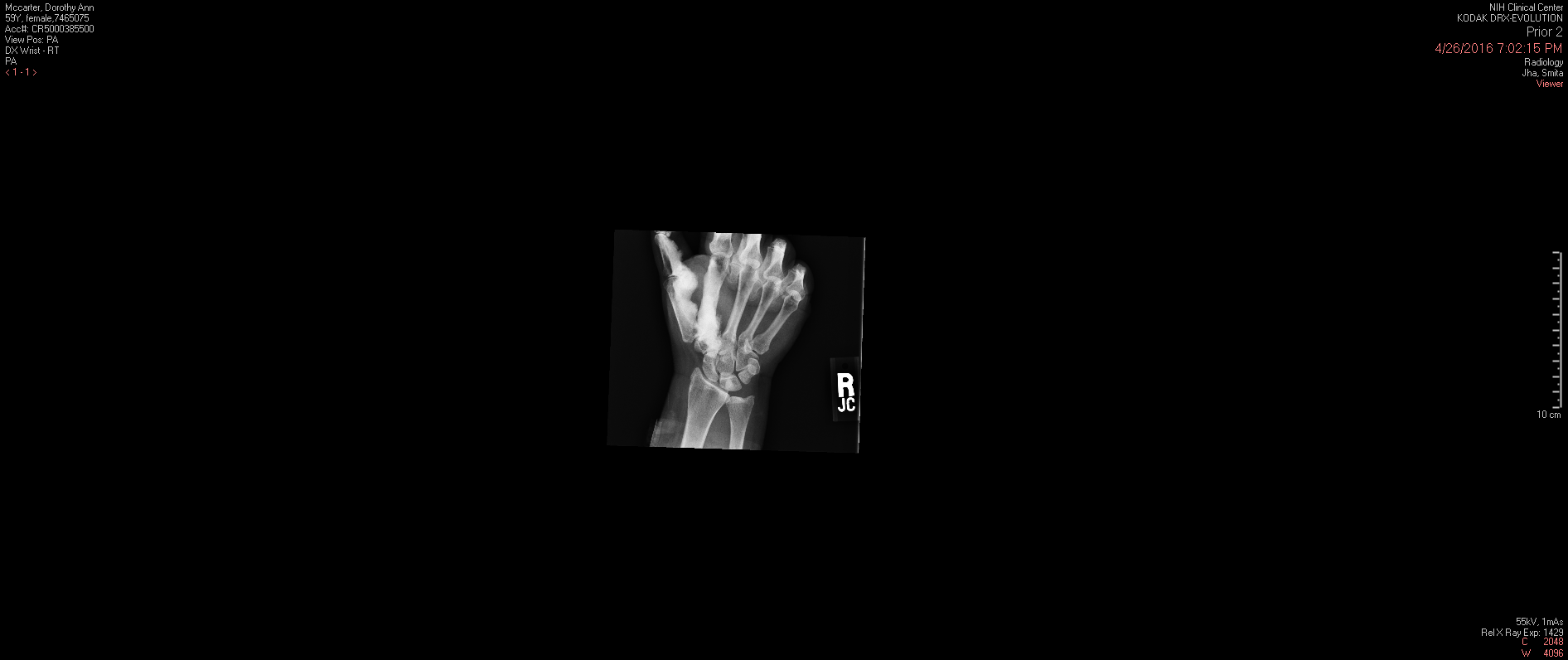

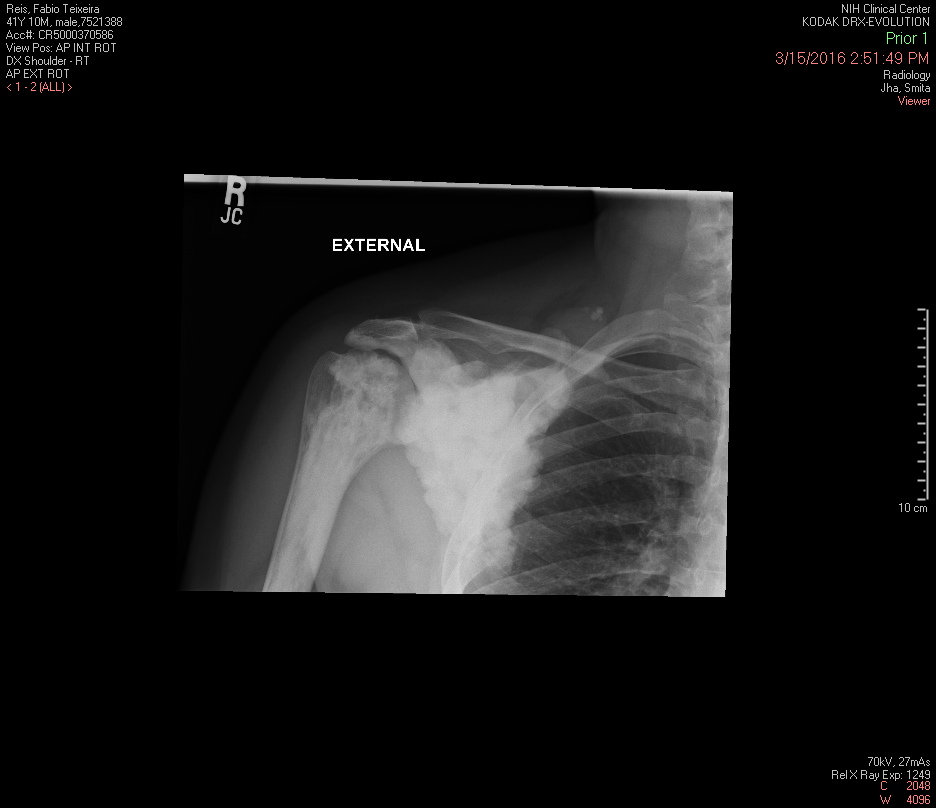


D

E

F

G

B

(**A-B)** Classic candle wax appearance of Melo-2 and Melo-4, respectively. (**C)** Myositis-ossificans like pattern of melorheostosis of Melo-9. (**D-G**) Classic candle wax appearance of Melo-9, Melo-16, Melo-18 and Melo-19, respectively. Note all but one patient has classic candle-wax appearance on radiographs.

Supplementary
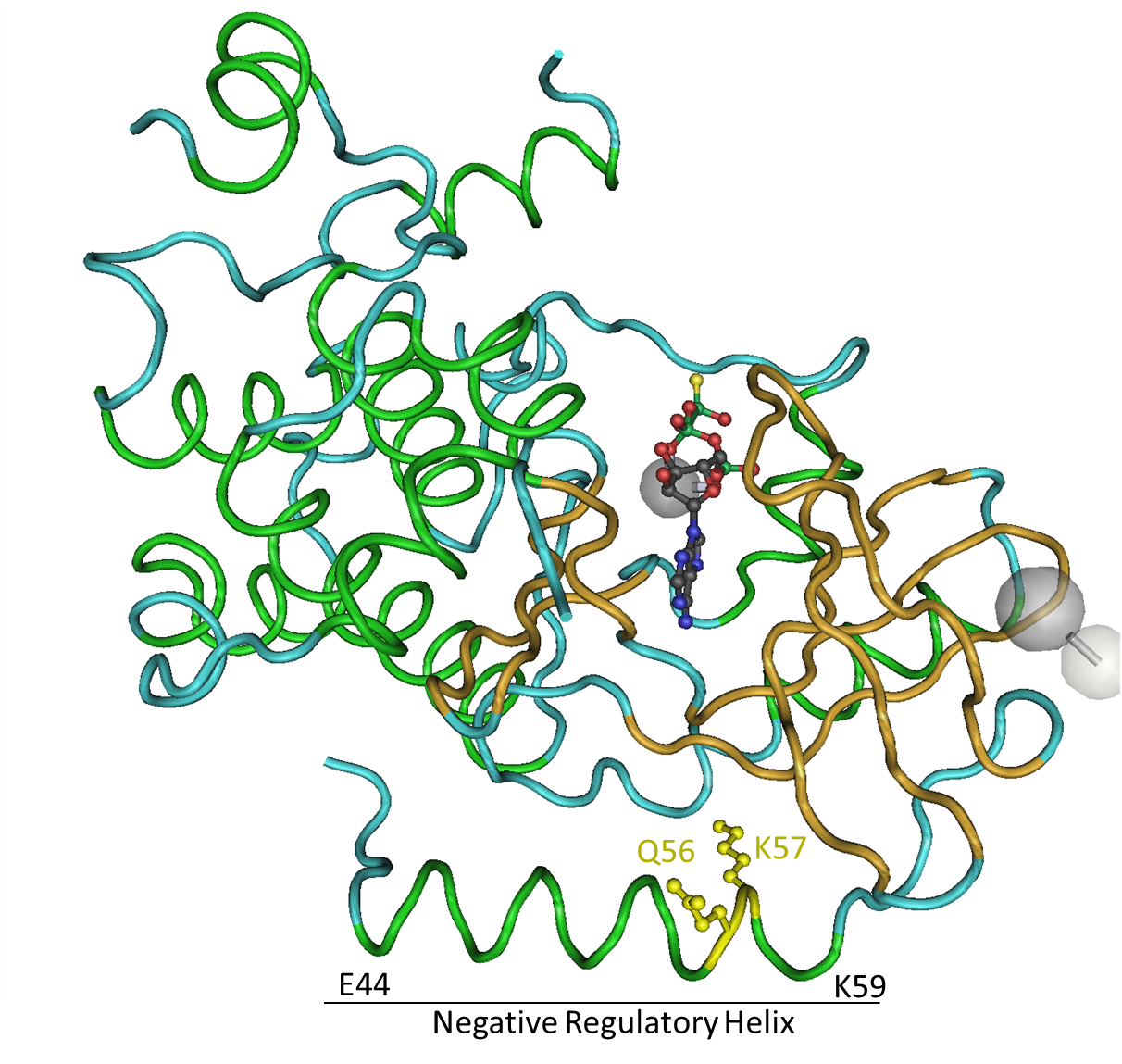
Figure 2. Molecular Model of MEK1 with Melorheostosis Associated Mutations Shown**.** The crystal structure of human MEK1^9^ rendered using Cn3D^10^ with the protein backbone in tube worm mode. Alpha helical structures are shown in green, beta sheet structures in gold, and disordered regions in cyan. The negative regulatory helix (amino acids 44-59) is marked, and the mutated amino acid residues in melorheostosis shown in yellow with side-chains rendered in ball-and-stick format.

Supplementary Figure 3. Immunohistochemistry of SW48 cells.


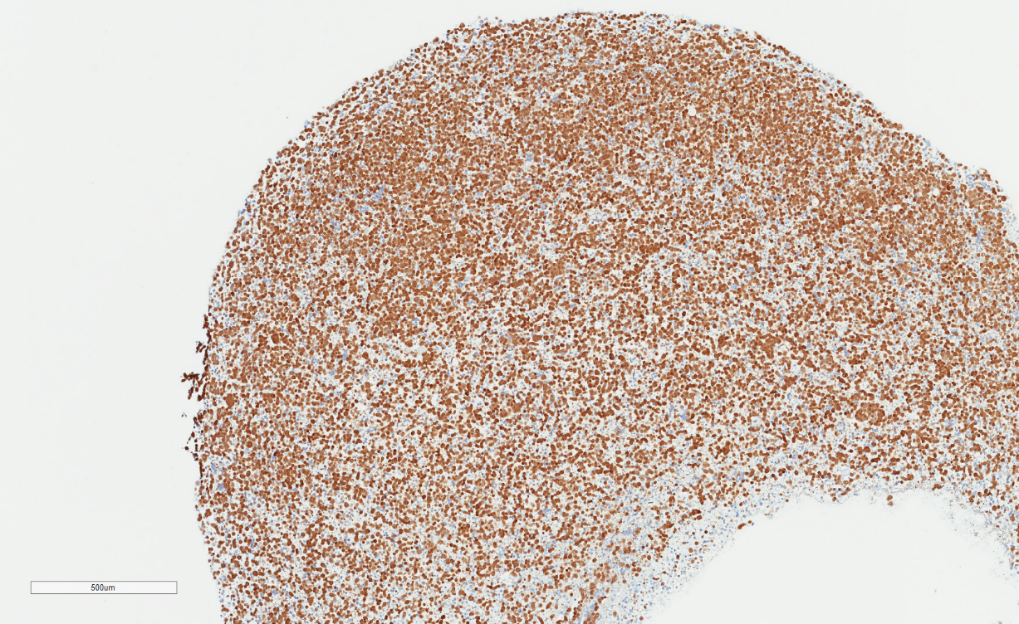

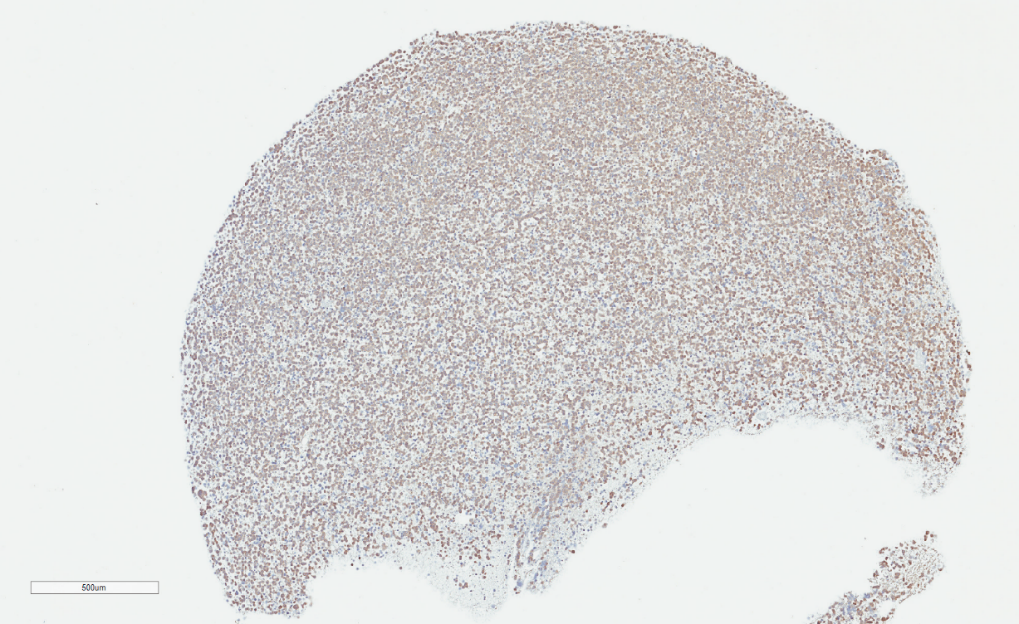


p-ERK1/2

IgG Isotype Control

SW48 cells, a colon cancer cell line known to harbor *MAP2K1* mutation p.K56P, were grown in culture and then a cell pellet was embedded in paraffin. Left panels shows SW48 cells stained with hematoxylin and the IgG isotype control antibody. Right panel shows SW48 cells stained with p-ERK1/2 antibody for activated ERK1/2 followed by HRP-conjugated secondary antibody. Brown staining indicates cells with p-ERK1/2.

Supplementary Figure 4. Activation of MEK1-ERK1/2 Pathway by *MAP2K1* Mutations***.***

Starv.

U0126

DMSO

***MAP2K1* p.K57N (Melo-18)**

Baseline

FBS stim.

Starv.

U0126

DMSO

Baseline

FBS stim.

p-ERK1/2

Tot ERK1/2

GAPDH


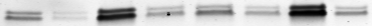

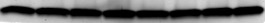

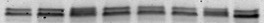


Unaffected Affected

| Melo-18 |  | Unaffected | |  |  | Affected |  |  |
| --- | --- | --- | --- | --- | --- | --- | --- | --- |
|  | Baseline | No stim. | DMSO | U0126 | Baseline | No stim. | DMSO | U0126 |
| p-ERK1/2 | 1.00 | 0.53 | 5.88 | 1.22 | 4.12 | 2.42 | 11.74 | 2.54 |


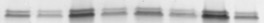

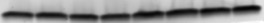

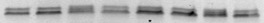


Starv.

U0126

DMSO

***MAP2K1* p.K57E (Melo-10)**

Baseline

FBS stim.

Starv.

U0126

DMSO

Baseline

FBS stim.

p-ERK1/2

GAPDH

Unaffected Affected

Tot ERK1/2

| Melo-10 |  | Unaffected | |  |  | Affected |  |  |
| --- | --- | --- | --- | --- | --- | --- | --- | --- |
|  | Baseline | No stim. | DMSO | U0126 | Baseline | No stim. | DMSO | U0126 |
| p-ERK1/2 | 1.00 | 0.35 | 3.53 | 0.91 | 1.02 | 0.46 | 3.45 | 0.78 |

Western blot analysis of osteoblast lysates from affected and unaffected bone of patients, Melo-18 (*MAP2K1* p.K57N, VAF 46%) and Melo-10 (*MAP2K1* p.K57E, VAF 10%). Band intensity of p-ERK1/2 was normalized against total ERK1/2 and shown in the tables.

Supplementary Figure 5. Similarity Between Melorheostosis and Injection Studies of Developing Limb.


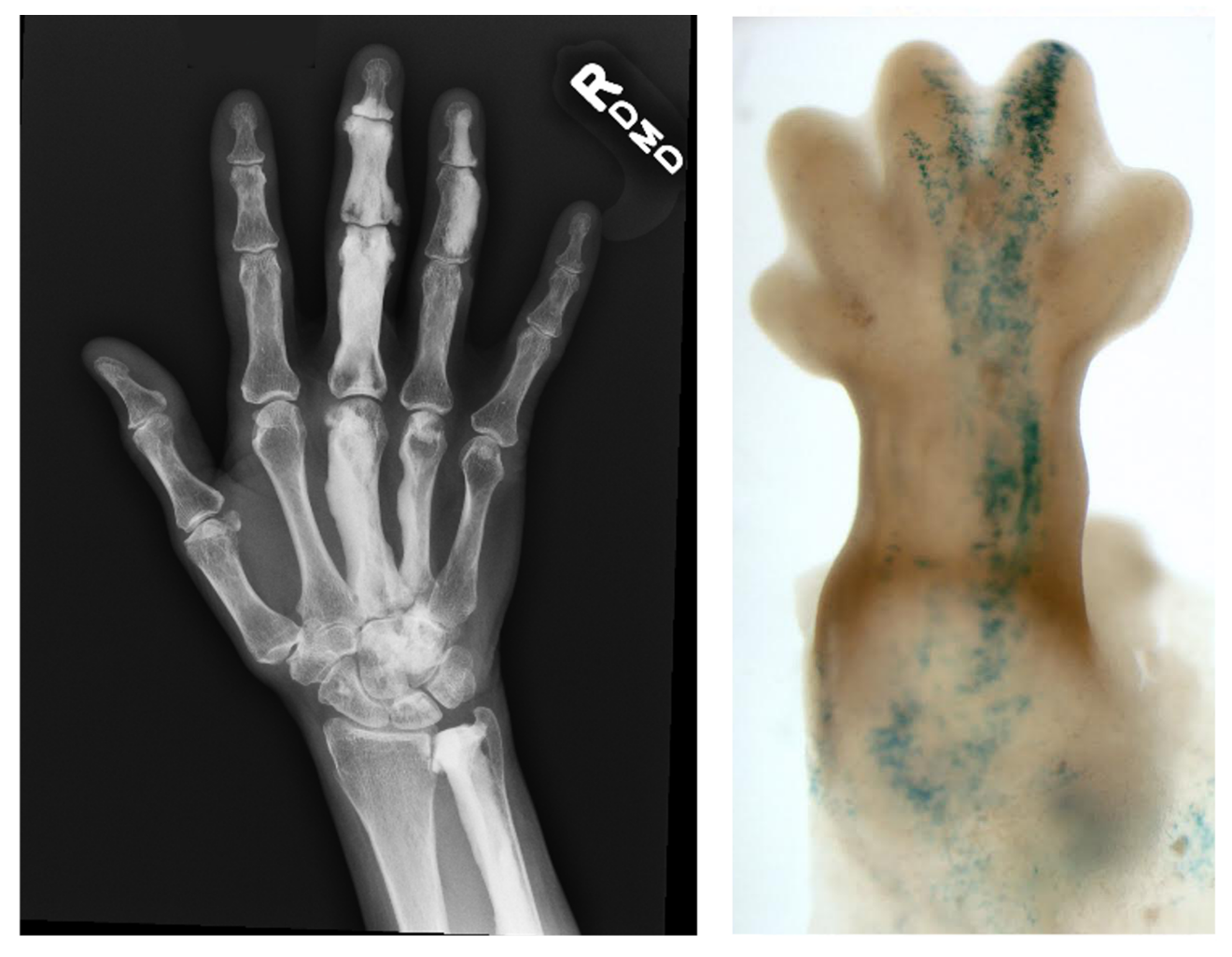


On the left, a patient with melorheostosis in the hand travels along the ulna and the long finger ray. Some melorheostosis is seen in the ring finger. On the right a developing mouse embryo has been genetically modified to produce LacZ reporter only in clones of developing cells^11^. Note the similar pattern. (Photograph curtesy Miguel Torres.)

# Supplementary Figure 6. Original Images of All Western Blots

## Supplementary Figure 6-1. Original images of Western blots for Fig. 4c

**
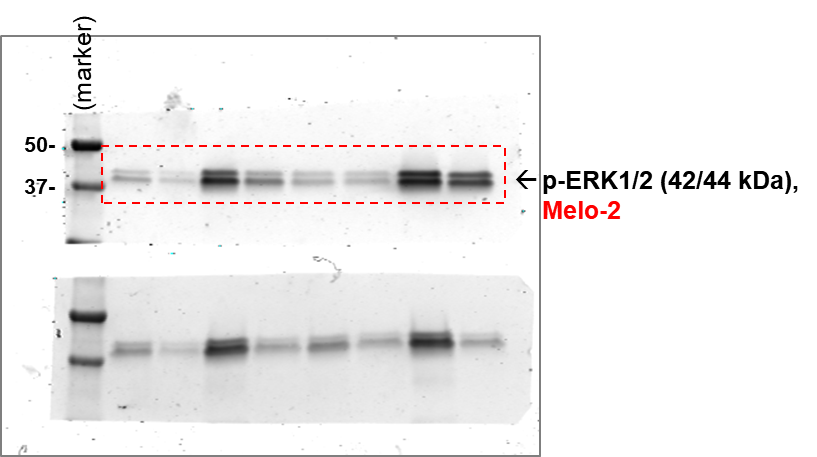

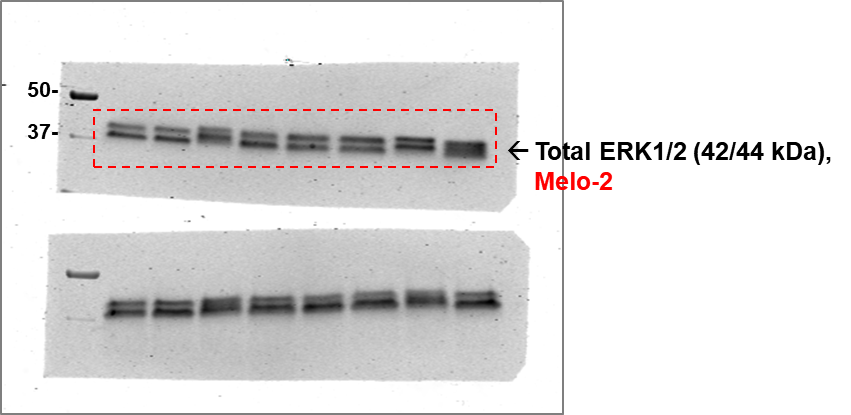

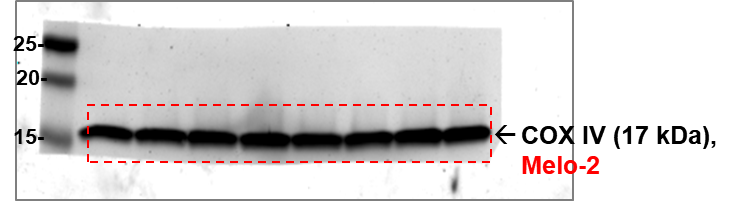
**

## Supplementary Figure 6-2. Original images of Western blots for Fig. 5b

**
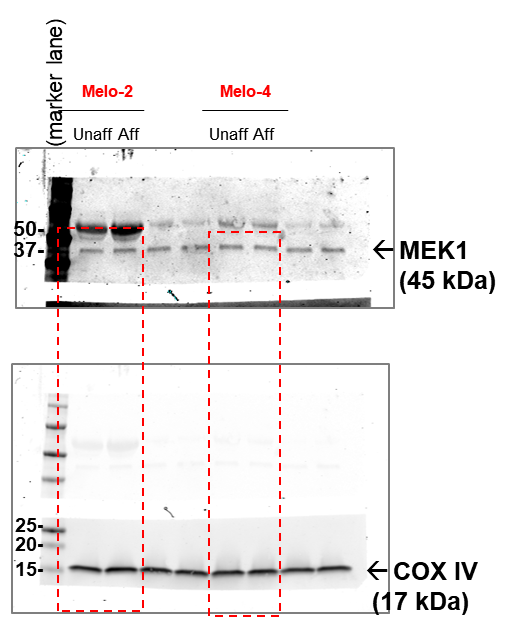

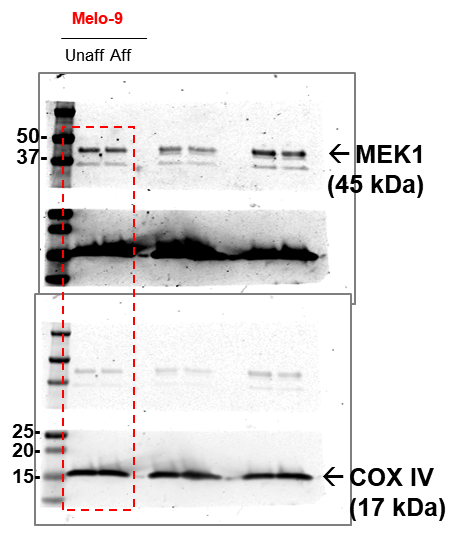

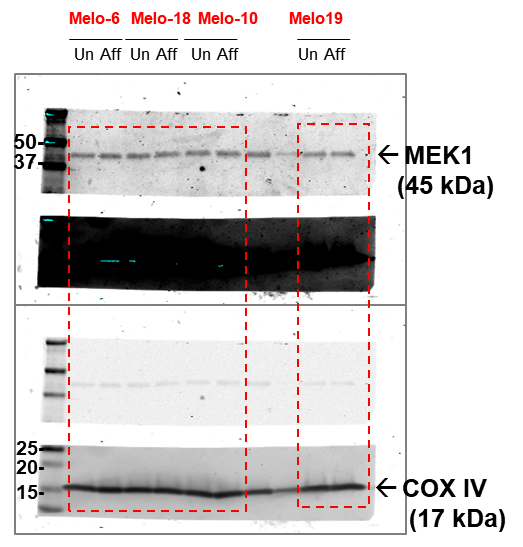
**

Supplementary Figure 6-3. Original images of Western blots for Fig. 5d**
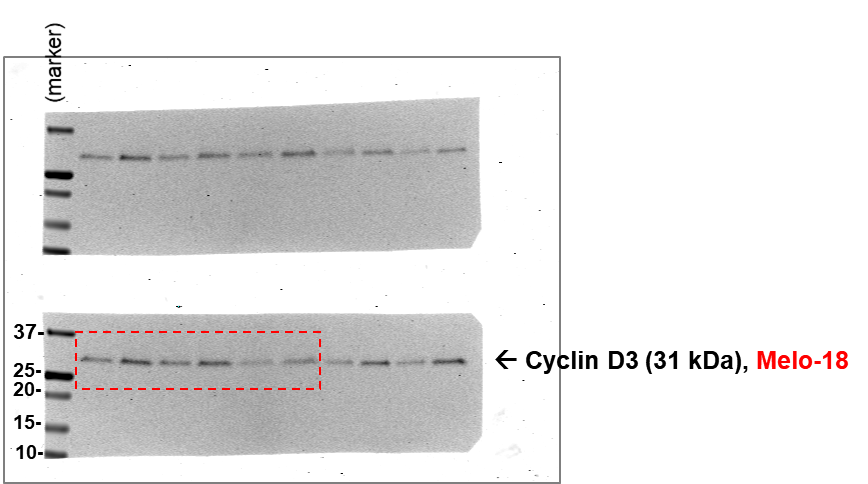

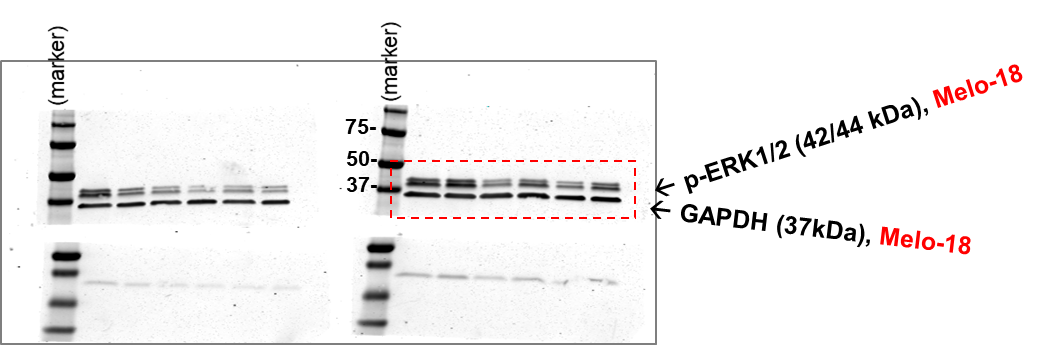
**

Supplementary Figure 6-4. Original images of Western blots for Supplementary Fig. 4a**
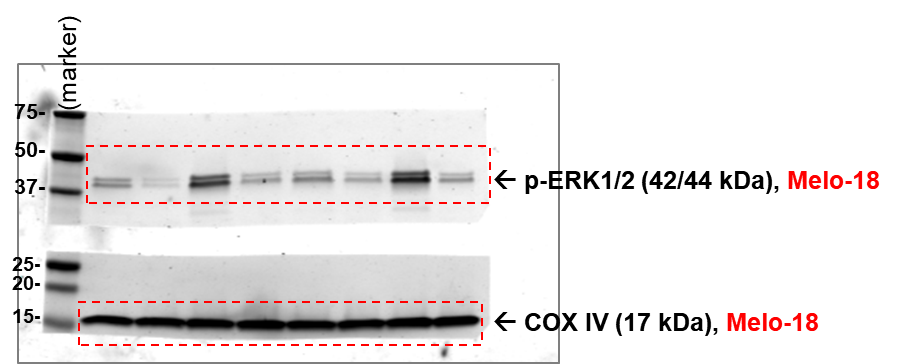
**

**
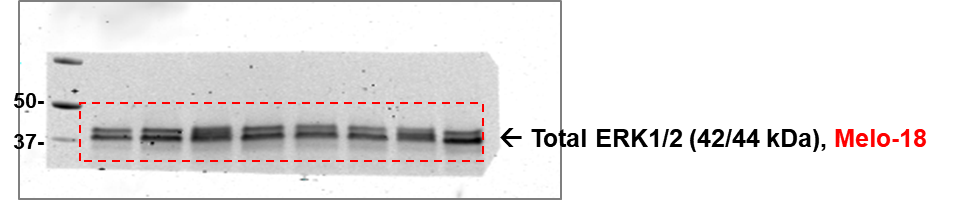
**

Supplementary Figure 6-5. Original images of Western blots for Supplementary Fig. 4b**
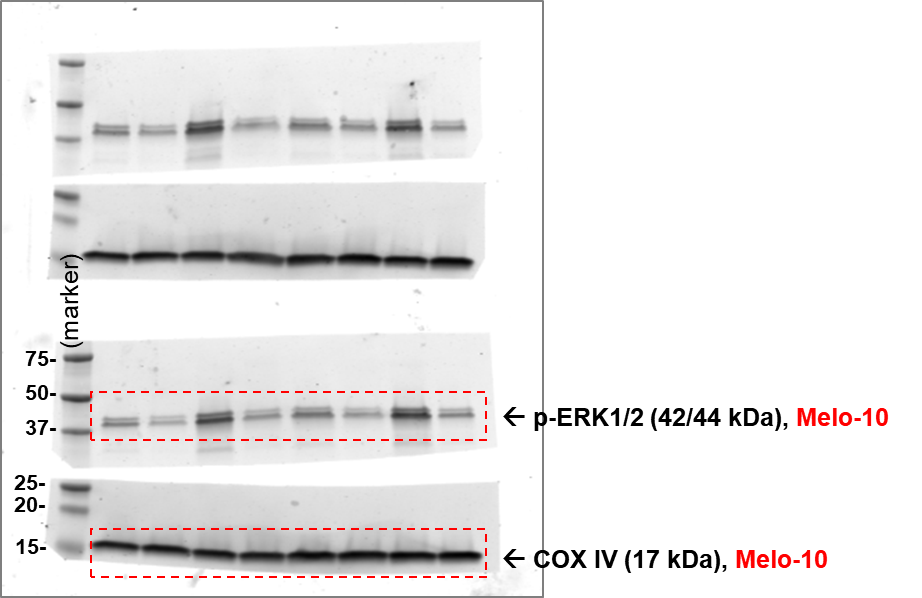

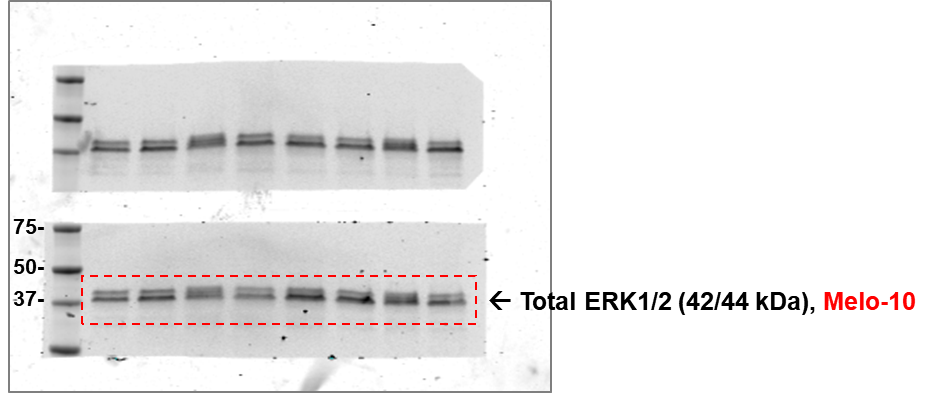
**

# Supplementary Figure 7. Gating strategy used for flow cytometry for pERK1/2 detection

SSC

-

A

SSC

-

A

Example of pERK1/2


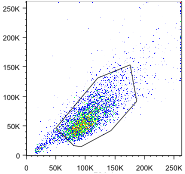


FSC/SSC


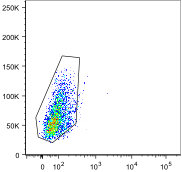


Live cells


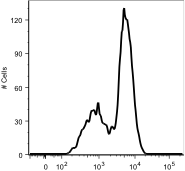


FSC-A LiveDead pERK1/2

# Supplemental References

1. Marks JL, Gong Y, Chitale D, et al. Novel MEK1 mutation identified by mutational analysis of epidermal growth factor receptor signaling pathway genes in lung adenocarcinoma. Cancer research 2008;68:5524-8.

2. Murugan AK, Dong J, Xie J, Xing M. MEK1 mutations, but not ERK2 mutations, occur in melanomas and colon carcinomas, but none in thyroid carcinomas. Cell Cycle 2014;8:2122-4.

3. Waterfall JJ, Arons E, Walker RL, et al. High prevalence of MAP2K1 mutations in variant and IGHV4-34-expressing hairy-cell leukemias. Nature genetics 2014;46:8-10.

4. Diamond EL, Durham BH, Haroche J, et al. Diverse and Targetable Kinase Alterations Drive Histiocytic Neoplasms. Cancer discovery 2016;6:154-65.

5. Arcila ME, Drilon A, Sylvester BE, et al. MAP2K1 (MEK1) Mutations Define a Distinct Subset of Lung Adenocarcinoma Associated with Smoking. Clinical cancer research : an official journal of the American Association for Cancer Research 2015;21:1935-43.

6. Chakraborty R, Hampton OA, Shen X, et al. Mutually exclusive recurrent somatic mutations in MAP2K1 and BRAF support a central role for ERK activation in LCH pathogenesis. Blood 2014;124:3007-15.

7. Couto JA, Huang AY, Konczyk DJ, et al. Somatic MAP2K1 Mutations Are Associated with Extracranial Arteriovenous Malformation. Am J Hum Genet 2017.

8. Trunzer K, Pavlick AC, Schuchter L, et al. Pharmacodynamic effects and mechanisms of resistance to vemurafenib in patients with metastatic melanoma. J Clin Oncol 2013;31:1767-74.

9. Fischmann TO, Smith CK, Mayhood TW, et al. Crystal Structures of MEK1 Binary and Ternary Complexes with Nucleotides and Inhibitors. Biochemistry-Us 2009;48:2661-74.

10. Wang Y, Geer LY, Chappey C, Kans JA, Bryant SH. Cn3D: sequence and structure views for Entrez. Trends Biochem Sci 2000;25:300-2.

11. Arques CG, Doohan R, Sharpe J, Torres M. Cell tracing reveals a dorsoventral lineage restriction plane in the mouse limb bud mesenchyme. Development 2007;134:3713-22.
